# Lipid nanoparticles (LNP) induce activation and maturation of antigen presenting cells in young and aged individuals

**DOI:** 10.1038/s42003-023-04555-1

**Published:** 2023-02-17

**Authors:** Jennifer Connors, David Joyner, Nathan J. Mege, Gina M. Cusimano, Matthew R. Bell, Jennifer Marcy, Bhavani Taramangalam, Kenneth M. Kim, Paulo J. C. Lin, Ying K. Tam, Drew Weissman, Michele A. Kutzler, Mohamad-Gabriel Alameh, Elias K. Haddad

**Affiliations:** 1 Drexel University College of Medicine, Department of Microbiology and Immunology, Philadelphia, PA USA; 2 Drexel University College of Medicine, Department of Medicine, Philadelphia, PA USA; 3 Drexel University College of Medicine, Department of Molecular and Cellular Biology, Philadelphia, PA USA; 4 Tower Health, Reading Hospital, Reading, PA USA; 5 Acuitas Therapeutics, Vancouver, Canada; 6 University of Pennsylvania, Perelman School of Medicine, Philadelphia, PA USA; 7 University of Pennsylvania, Penn Institute for RNA Innovation, Philadelphia, PA USA

**Keywords:** Innate immune cells, Adjuvants

## Abstract

Herein, we studied the impact of empty LNP (eLNP), component of mRNA-based vaccine, on anti-viral pathways and immune function of cells from young and aged individuals. eLNP induced maturation of monocyte derived dendritic cells (MDDCs). We further show that eLNP upregulated CD40 and induced cytokine production in multiple DC subsets and monocytes. This coincided with phosphorylation of TANK binding kinase 1 (pTBK1) and interferon response factor 7 (pIRF7). In response to eLNP, healthy older adults (>65 yrs) have decreased CD40 expression, and IFN-γ output compared to young adults (<65 yrs). Additionally, cells from older adults have a dysregulated anti-viral signaling response to eLNP stimulation, measured by the defect in type I IFN production, and phagocytosis. Overall, our data show function of eLNP in eliciting DC maturation and innate immune signaling pathways that is impaired in older adults resulting in lower immune responses to SARS-CoV-2 mRNA-based vaccines.

## Abstract (web summary)

The role of empty lipid nanoparticles in eliciting dendritic cell maturation and innate immune signaling is shown to be impaired in older adults, potentially contributing to lower immune responses to SARS-CoV-2 mRNA-based vaccines.

## Introduction

Two of the current COVID-19 vaccines are based on nucleoside-modified messenger RNA (mRNA)-lipid nanoparticles (LNP). This vaccine platform has been widely tested in preclinical and clinical studies showing its effectiveness in generating protective humoral immunity^1–7^. Additionally, it has been shown that the immune stimulatory effects of the mRNA-LNP vaccine platform is not due to the inclusion of nucleoside modified mRNA but by the intrinsic adjuvant effect of the ionizable lipid, a major component of the LNP formulation^1,8^. However, the mechanism of this response has not been fully investigated. Previous studies seeking to elucidate the mechanism of these immunomodulatory agents has identified the involvement of different pathogen-associated molecular pattern (PAMP)-sensing receptors and pathways in mice^1,8–11^ but few, if any, studies exist in humans. Recently, Alameh et. al published that the addition of empty LNP (eLNP) not only intrinsically promoted the secretion of IL-6, among other cytokines in mice, but also induced robust T_FH_ cell responses after immunization. Removal of the ionizable lipid from the LNP abrogated the vaccine response highlighting its essential role in adjuvanticity. Another group recently published that lipid-formulated RNA vaccines induce production of IL-1 which then induce pro-inflammatory cytokines like IL-6^10^. More studies are needed to determine how the LNP is sensed and how pro-inflammatory cytokines and chemokines including IL-6 production are regulated to guide the design of better lipid-based adjuvant, improve the current mRNA-LNP vaccine platform, and understand mechanisms.

Age is another factor that can influence vaccine response. The progressive decline in the function of the immune system with increasing age is a condition known as immunosenescence which not only leads to decreased protection against infectious pathogens but also leads to the inability to mount protective immune responses following vaccination^12^. An age associated decrease in protection against infectious pathogens is demonstrated by the increased mortality rates in individuals above 65 years of age following infection. For instance, individuals aged 65–74 were upwards of 95 times more likely to die from SARS-CoV-2 infection when compared to the 18–29-year-old population^13^. With the global population over the age of 65 expected to double by the year 2050^14^, it is imperative to gain a deeper understanding of the underlying mechanisms of immunosenescence and mechanism of action of different adjuvants (e.g., materials and/or formulations) to effectively protect this portion of the population.

In those over 65, it has been shown that there are deficits in innate antiviral signaling and adaptive immune response to the SARS-CoV-2 vaccine particularly for variants of concern, suggesting that LNP stimulatory effects could be altered in the aged^15^. Many of the recent studies that look at SARS-CoV-2 vaccination in people over 65 show induction of strong, but rapidly declining, humoral responses against the virus, and its variants^16^. Other studies show that coordination of SARS-CoV-2 antigen-specific responses are disrupted in those individuals^17^. The data suggests less of a role for the humoral response than SARS-CoV-2 specific CD4^+^ or CD8^+^ T cells in providing complete protection against severe disease in older adults. Understanding how lipid nanoparticles interact with the innate immune system, especially within an aging context can help the development of adjuvants to induce stronger and more durable responses in older individuals.

In this report, we demonstrate that the eLNP formulation used in previous studies in mice^1,2^ is effective at maturing monocyte-derived dendritic cells and activating DCs and monocytes from human PBMCs causing secretion of not only innate immune cytokines and chemokines but also pro-T_FH_ cytokines including IL-21 and IL-6^18^. We also demonstrate that there is an age-specific difference between important innate immune cytokines and chemokines and show that eLNP initiate TGF-β production as a potential mechanism of human T_FH_ cell differentiation^19^. Mechanistically, the capacity of eLNP to elicit robust induction of innate and adaptive responses shows evidence of being dependent on pathways that converge upon TBK-1 (phosphorylation of TBK-1), and/or IRF-7 though differences between younger and older adults was less noticeable. We also show that the addition of eLNP acts as a stimulator of phagocytosis. This study sheds light on the mechanism of the immunomodulatory component of the recent SARS-CoV-2 vaccines and how we can improve vaccine efficacy in the more vulnerable population.

## Results

### eLNP treatment promotes robust innate immune response and DC maturation

**Fig. 1:**
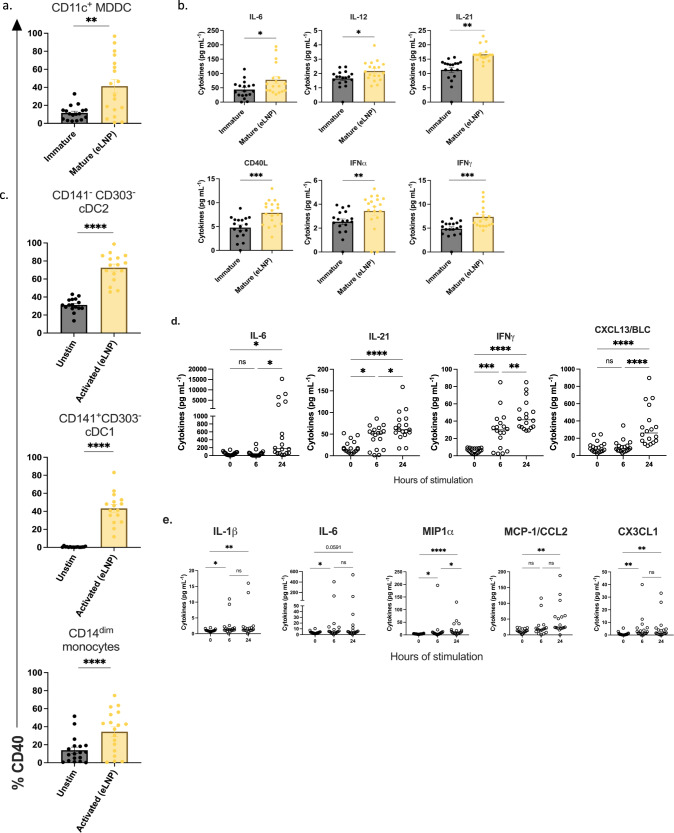
eLNP treatment promotes robust innate immune response and maturation. **a** Activation status of monocyte-derived DCs as measured by CD40 expression after 24 h of 15 μg/mL (total lipids, or~7.5 μg/mL ionizable lipid) empty LNP (eLNP) stimulation (yellow). *n* = 18. **b** Innate cell cytokine expression after 24 h eLNP stimulation (yellow) compared to that in unstimulated (media) cells (grey) of MDDCs. *n* = 18 for each group as measured by Luminex. **c** Activation status of DC and monocyte subsets from PBMCS as measured by CD40 expression after 24 h of eLNP stimulation (yellow). *n* = 18. **d** Dynamics of cytokines from PBMCS at 0, 6, and 24 h after eLNP stimulation. *n* = 18 as measured by Luminex. **e** Dynamics of cytokines from monocytes at 0, 6, and 24 h after eLNP stimulation. *n* = 18 as measured by Luminex. Each individual circle represents one individual subject. Data was analyzed either using non-parametric Mann Whitney T-test or by one-way ANOVA followed by Tukey’s test. *P < 0.05, **P < 0.01, ***P < 0.001, ****P < 0.0001.

The effect of eLNP on innate immune cells is not fully elucidated in humans. To investigate this, we tested the ability of eLNP to induce DC maturation in vitro. Monocytes isolated from healthy participant PBMCs (*n* = 18; age range 24–75 yrs) were treated with GM-CSF/IL-4 for 48 h followed by maturation with a dose of 15 μg/mL (total lipids, or~7.5 μg/mL ionizable lipid) empty LNP (eLNP) for 24 h. We assessed the frequency of surface costimulatory and HLA marker-expressing cells in eLNP-treated MDDCs compared to unstimulated cells after 24 h. The gating strategy used for MDDCs, and the determination of their surface marker frequencies is shown in Supplemental Fig. 1. We found that eLNP significantly up-regulated the percentage of CD40-positive human MDDCs compared to unstimulated control (*p* = 0.0043) (Fig. 1a). We also see an upregulation in the maturation markers of MDDCs (CD83 (*p* = 0.0001), CD86 (*p* = 0.0114), and HLA-DR (*p* = 0.002) (Supplementary Fig. 2a, b). We also tested the production of cytokines following in vitro DC maturation by eLNP. IL-6 (*p* = 0.0321), IL-12 (*p* = 0.0294), IL-21 (*p* < 0.0001), CD40L (*p* = 0.0009), IFNα (*p* = 0.0040), and IFNγ (*p* = 0.0008) significantly increased after 24 h of eLNP stimulation (Fig. 1b). Thus, eLNP can induce pro-T_FH_ cytokines as well as key cytokines and chemokines that are efficient in activating innate immune response like IFN-α and IFN-γ. In comparison, traditional lipid-based adjuvants such as AS01 also strongly induced IFNγ and related genes in the signaling pathway that led to the recruitment of CD11b^+^ Ly6G^Hi^ neutrophils, CD11b^+^ Ly6C^Hi^ monocytes and CD11c^+^MHCII^Hi^ DCs and upregulation of DC maturation markers (CD40, CD86, CD80) compared with non-adjuvanted mice^20^. Agonists for TLR7/8, RIG-I, and STING signaling pathways have also been shown to strongly induce anti-viral immunity (IFN-α and IFN-γ), as well as induction of phagocytosis^21^. All together, these data using human MDDCs in our in vitro culture system confirm that eLNP can induce the maturation of human MDDCs and may a play a role in the initiation of innate immunity and a further role in T_FH_ differentiation, function, and proliferation^22^.

We further assess the impact of eLNP on multiple DC and monocyte subsets using multiparametric flow cytometry. Specifically, we determined the frequency of surface costimulatory marker-expressing cDC1, cDC2, and monocytes following eLNP treatment of PBMCs (Fig. 1c). To achieve this, PBMCs were stimulated with eLNP for 24 h and then DCs and monocytes subsets were analyzed for the expression of surface marker and cytokine secretion. Conventional type 1 DCs (cDC1) are the primary subset that cross-presents antigen to CD8^+^ T cells and predominantly produce IL-12^23–26^. In contrast, conventional type 2 DCs (cDC2) have been associated with CD4^+^ T_FH_ cell responses including GC T_FH_ responses^22,26,27^. Additionally, we monitored CD14^+^CD16^−^, CD14^+^CD16^+^ and CD14^dim^CD16^+^ monocytes. The latter plays a vital role in antiviral immunity and response to vaccination by patrolling the vascular endothelium in response to viral exposure and produce TNF-α and IL-1b^28,29^. We found that eLNP induces the expression of CD40 in cDC2 (*p* < 0.0001) and cDC1 (*p* < 0.0001), and CD14^dim^ CD16^+^ monocytes (*p* = 0.0004). Additionally, we also see the upregulation of multiple activation markers on monocyte and DC subsets from PBMCS, namely OX40L (cDC2 (*p* < 0.0001), cDC1 (*p* = 0.0174)) (Supplementary Fig. 3b). We also examined the eLNP-induced cytokine and chemokine profile of PBMCs. After stimulation with eLNP over 0-,6-, and −24 h, the production of cytokines and chemokines was significantly elevated consistent with a pro-inflammatory phenotype compared to unstimulated cells, namely IL-6 (*p* = <0.0001), IL-21 (*p* < 0.0265), IFNγ (*p* < 0.0001), and CXCL13 (*p* = 0.0001) (Fig. 1d). To further investigate how eLNP might affect a first line defense, we isolated monocytes (CD14 + ) from PBMCs and stimulated with eLNP at 0-,6-, and –24 h and measured the production of cytokines and chemokines. We found that monocytes had significantly increased upregulation of IL-1β (*p* = 0.0132), IL-6 (*p* = 0.0208), and CX3CL1 (*p* = 0.0040) by 6 h that was sustained through 24 h (Fig. 1e). The monocyte chemokines MIP1α and MCP-1 were significantly upregulated by 24 h post stimulation (MIP1α (*p* < 0.0001) and MCP-1 (*p* < 0.0001)). These data confirm that eLNP activates DCs and monocyte subsets and suggest the possibility that eLNP may mediate its effect by activation and maturation of innate immune cells.

### TBK-1/ IRF7 axis is induced by eLNP

**Fig. 2:**
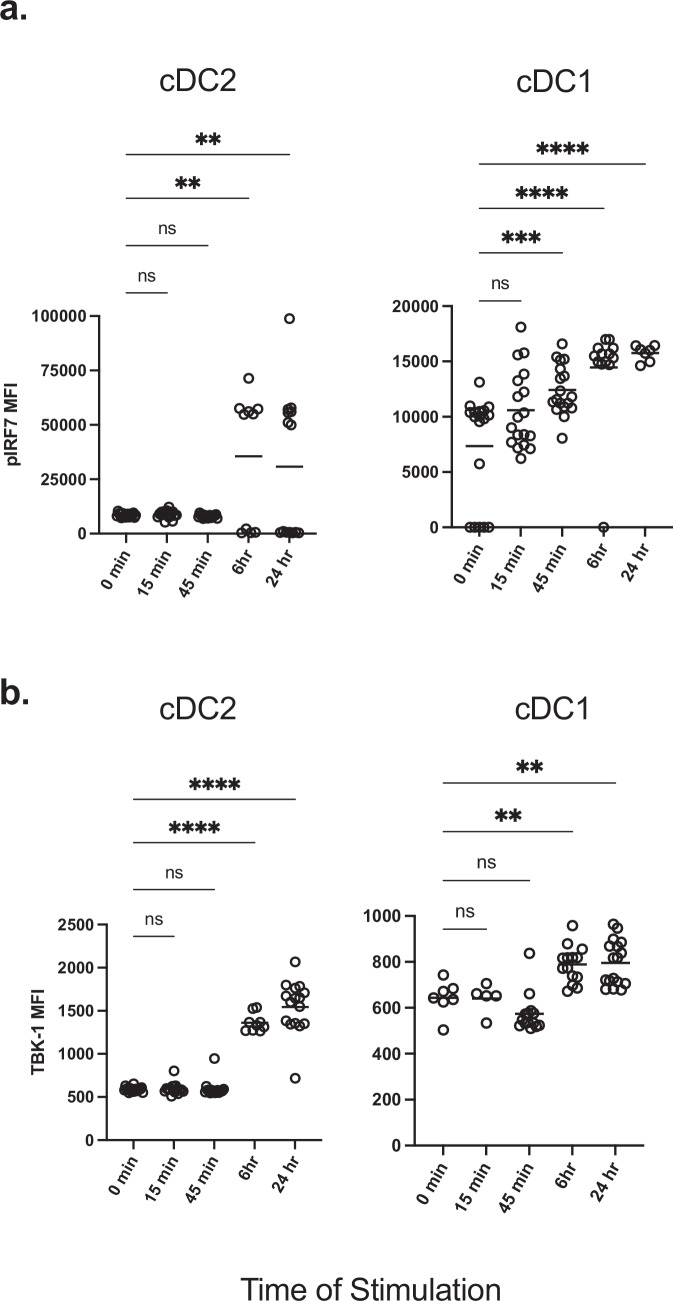
IRF7/TBK-1 axis is important for eLNP-induced innate response. Human PBMCs from healthy donors were either stimulated with 15 μg/mL (total lipids, or ~7.5 μg/mL ionizable lipid) eLNP for 15 min, 45 min, 6, or 24 h. For unstimulated and control conditions refer to Fig. 1. Cells were permeabilized, fixed, and stained for (**a**) phosphorylated interferon response factor 7 (IRF7) transcription factor or (**b**) phosphorylated TBK-1. After gating on monocyte and DC subsets, the geometric mean intensity (MFI) was measured using phosflow cytometry. Each individual circle represents one individual subject. *P < 0.05, **P < 0.01, ***P < 0.001, and ****P < 0.0001 by one-way ANOVA.

DCs and monocytes help shape the innate immune response via the activation of pattern recognition receptors (PRR) and other molecular sensors. The activation of different TLR (such as TLR 2,4, and 7/8) and RLR pathways initiates a series of cross-talk signaling events that are mediated by TBK-1, which leads to the phosphorylation, dimerization, and translocation of the transcription factor, IRF7^30,31^. Next, we determined if eLNP can elicit phosphorylation and activation of IRF7 and its upstream initiator, TBK-1. Human PBMCs from 18 donors were stimulated with a dose of 15 μg/mL (total lipids, or~7.5 μg/mL ionizable lipid) eLNP for 15 min-, 45 min-, 6-, and 24 h, and flow cytometry was performed to determine whether eLNP alone can induce and activate IRF7 in DC and monocyte subsets, as measured by phosphorylation of IRF7 (pIRF7). Overall, eLNP treatment was able to notably induce IRF7 activation in cDC2 and cDC1 (Fig. 2). Following a 6-hour stimulation, significant upregulation of pIRF7 was observed in cDC2 DCs (*p* = 0.0022) and was maintained through 24 h of stimulation (p = 0.0067) (Fig. 2a). Significant upregulation of pIRF7 was observed as early as 45 min in cDC1 DCs (*p* = 0.0009), and this significant upregulation was enhanced and then maintained for both the 6- and 24-hour stimulations, respectively (6 h *p* = <0.0001; 24 h *p* = <0.0001). In addition to the phosphorylation of IRF7, we also saw phosphorylation of TBK-1 (pTBK-1) after eLNP stimulation in both cDC1 and cDC2 DCs (Fig. 2b). Here, we saw a significant upregulation of pTBK-1 following a 6 h stimulation in both cDC2 (*p* = 0.0052) and cDC1(p < 0.0001) DCs. These increased levels of pTBK-1 were maintained through a 24 h stimulation for both DC subsets (cCD2, *p* = 0.0067; cDC1, *p* = <0.0001). We did not see a significant difference in pIRF7 or pTBK-1 induction in CD14^dim^ monocytes (Supplemental Fig. 4). Overall, these results demonstrate that eLNP can activate IRF and TBK-1 signaling pathways.

### Phagocytosis is induced in cDC2, cDC1 in response to eLNP stimulation

**Fig. 3:**
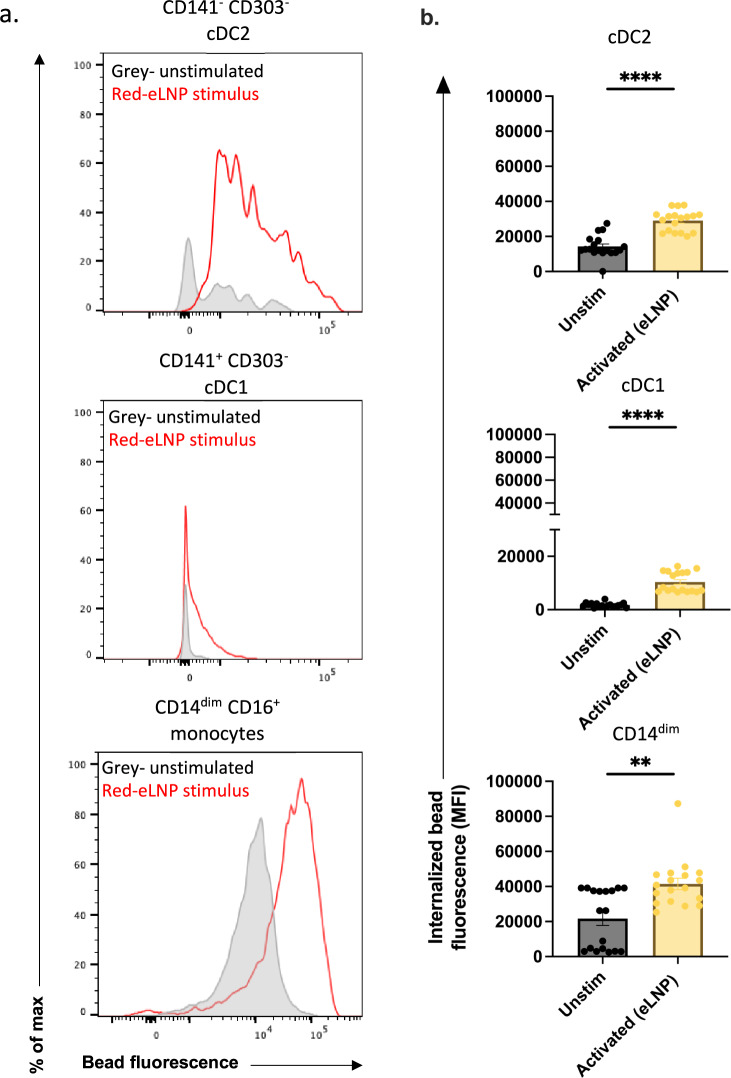
Phagocytosis is induced in cDC2, cDC1, CD14^dim^ CD16^+^ monocytes in response to eLNP stimulation. Phagocytosis measured using fluorescent beads and multiparametric flow cytometry. PBMCs were incubated overnight with stimulus (15 μg/mL (total lipids, or ~7.5 μg/mL ionizable lipid) eLNP or plain medium) and with beads for a further 3 h. **a** FACS plot examples of phagocytosis assay as geometric mean fluorescent intensity (MFI) by flow cytometry. **b** Phagocytosis after 24 h eLNP stimulation (yellow) compared to that in unstimulated cells (grey) of PBMCS. *n* = 18 for each group. Each individual circle represents one individual subject, *n* = 18. *P < 0.05, **P < 0.01, ***P < 0.001, and ****P < 0.0001 by or non-parametric Mann Whitney T-test.

A primary function of innate immune cells is phagocytosis. Here, we investigated whether eLNP can induce phagocytosis in antigen presenting cells utilizing an in vitro phagocytosis assay which measures the uptake of fluorescent beads. We stimulated healthy adult PBMCs with eLNP for 24 h and flow cytometry was performed to analyze the mean fluorescence intensity (MFI) of engulfed particles as a function of phagocytosis of DC subsets and CD14^dim^ monocytes. Upon stimulation with eLNP, cDC2, cDC1 subsets show increased fluorescence (cDC2, *p* < 0.0001; cDC1, *p* < 0.0001) compared to unstimulated indicating superior phagocytic function following stimulation with eLNP (Fig. 3a, b). CD14^dim^ monocytes show increased fluorescence when stimulated indicating greater activation (*p* < 0.01) (Fig. 3b).

### eLNP elicits TGF-β production in PBMCs and MDDCs

**Fig. 4:**
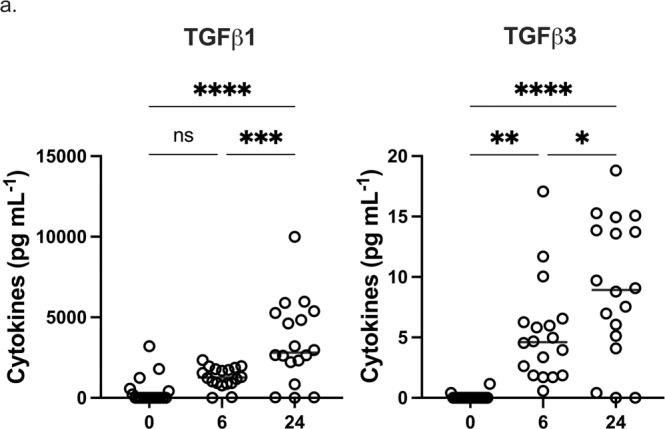
eLNP treatment promotes TGF-B secretion. **a** Dynamics of TGF-β secretion from PBMCS at 0, 6, and 24 h after eLNP stimulation. *n* = 18. Data were combined from at least two independent experiments. Each individual circle represents one individual subject. Data are from one independent experiment (**a**) One-way ANOVA followed by Tukey’s test was applied in **a**. *P < 0.05, **P < 0.01, ***P < 0.001, ****P < 0.0001.

TGF-β has previously been shown to play essential roles in the differentiation and function of T cells, inhibiting the differentiation of Th1 and Th2 T cells^32^ while promoting Treg, Th17, and T_FH_ differentiation^19,32^. To test whether eLNP treatment leads to production of TGF-β isoforms (TGF-β1, TGF-β2, and TGF-β3) within innate immune cells, PBMCs were treated for 6 or 24 h with eLNP (15 μg/μL-1 total lipids, or 7.5 μg/μL-1ionizable lipid) and compared to both unstimulated cells. The levels of TGF-β1 in PBMCs were induced after 24 h of stimulation (*p* = <0.0001) while the levels of TGF-β3 were induced starting at 6 h (*p* = 0.0011) and increased when measured after 24 h of stimulation (*p* = <0.0001) (Fig. 4a). Similarly, MDDCs from donors were treated for 24 h with eLNP and compared to unstimulated cells. Interestingly, TGFβ2 levels were downregulated upon stimulation with eLNP for 24 h in MDDCs (*p* = 0.0065) suggesting a differential role between cell types (supplemental Fig. 5a).

### eLNP alter MDDC maturation and cytokine secretion in younger and older adults

**Fig. 5:**
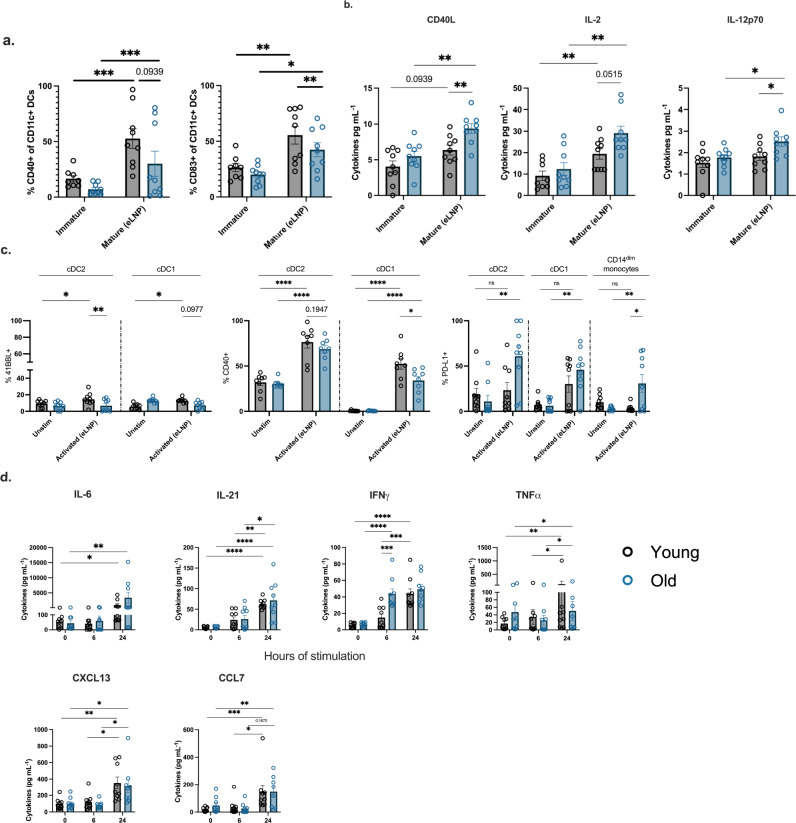
eLNP treatment promotes monocyte-derived DC maturation and the secretion of pro-TFH cytokines that are also diminished between young and older participants. **a**–**d** Monocyte-derived dendritic cells (MDDCs) differentiated in vitro from monocytes of healthy human PBMCs from older and younger donors, were treated with 15 μg/mL (total lipids, or ~7.5 μg/mL ionizable lipid) eLNP for 24 h and compared to unstimulated cells (just media) as controls. MDDCs were harvested and stained for surface HLA and costimulatory molecule expression and analyzed by flow cytometry. Values are shown as the frequencies of (**a**) CD83, (**b**) CD40, (**c**) CD86 (**d**) HLA-DR in the live, CD3- CD19- CD11c + MDDC population. Supernatants were also harvested, and selected cytokines were measured with Luminex after 24 h of stimulation (**a**–**d**). Nine samples per group were measured, except 24 h LPS where only three samples per group were analyzed. Results are expressed as mean ± SEM of 2 independent experiments (*n* = 9). Each individual circle represents one individual subject. *P < 0.05, **P < 0.01, ***P < 0.001, and ****P < 0.0001 by one-way ANOVA or non-parametric Mann Whitney T-test.

After establishing that eLNP can induce an immune response, we aimed to describe how eLNP would affect an aged immune system. Therefore, we stratified our 18 subjects as young and old. Our old volunteers are healthy, non-frail individuals enrolled into the study are accrued with an equal sex distribution. Individuals with comorbid conditions like cancer within the last 5 years, or other immunocompromising conditions, and steroid use were excluded. Inclusion criteria included controlled hypertension, occasional aching joints from arthritis and not taking daily nonsteroidal anti-inflammatory drugs or acetaminophen, and controlled diabetes. The average age for adults was 30 years (range 24–36 years) whereas for older group it was 73 years (range 67–83 years) (Supplemental Table 1). Utilizing our cohort of 9 younger adults (< 65) and 9 older adults (> 65) we analyzed the effect of eLNP on the maturation and activation of the innate immune system. We found that eLNP significantly up-regulated the percentage of CD40- and CD83-positive human MDDC compared to unstimulated control (young, *p* = 0.0079; old, *p* = 0.0478) (Fig. 5a–d) for both older and younger participants. Specifically, the increase in eLNP-mediated CD83 expression was noted to be significantly different in older and younger participants (*p* = 0.0041) (Fig. 5a). Empty LNP induced the expression of CD40 (Fig. 5a) in both younger (*p* = 0.0006) and older participants, which is known to be important for human T_FH_ helper function via CD40L engagement. However, intergroup comparison showed that CD40 expression was higher for younger participants but did not reach statistical significance (*p* = 0.0939) (Fig. 5a). We also examined the eLNP-induced cytokine and chemokine profile of MDDCs within this aged cohort. Empty LNP administration significantly increased the secretion of CD40L (young = 0.0939, old *p* = 0.0086), IL-2 (young = 0.0015, old *p* = 0.0031), and IL-12 (old *p* = 0.0151) (Fig. 5b). Namely, we saw significantly more cytokine production in older vs younger adults in CD40L (*p* = 0.0061), IL-2 (*p* = 0.0515), and IL12p70 (*p* = 0.0376). Overall, our results show that eLNP can induce the maturation of MDDCs in vitro in both young and old individuals, however maturation markers are significantly lower in older subjects whereas pro-inflammatory cytokines are higher.

### eLNP induces age-specific activation in cDC1, cDC2, and CD14^dim^CD16^+^ monocytes from PBMCs

To determine the effect that eLNP has on the activation of monocyte and DC subsets between age groups, PBMCs from healthy young or older participant were treated with either eLNP for 6 or 24 h. We assessed the frequency of surface costimulatory marker-expressing cells in eLNP-treated PBMCs compared to unstimulated cells after 24 h (Fig. 5c). 41BBL is expressed on antigen-presenting cells that, upon ligation to its cognate receptors, activates both CD4 and CD8 T cells and induces their proliferation^33,34^. We found that that 41BBL was upregulated with eLNP stimulation in cDC2 (p = 0.0283) and cDC1 (*p* = 0.0090) of younger donors. We also see an age specific difference between young and older participants in cDC2 when stimulated with eLNP (*p* = 0.0019) and a trend in cDC1 (*p* = 0.0977). We also observed a significant upregulation in the expression of CD40 due to eLNP stimulation in the same cell types cDC1 (young, *p* < 0.0001; old, *p* = 0.0004) and cDC2 (young, *p* < 0.0001; old, *p* < 0.0001). PD-L1 engagement limits the proliferation of antigen-specific T cells in the germinal center by binding to activated T cells, B cells, and other myeloid cells. We found that in response to eLNP stimulation, only cDC2 (*p* = 0.0002), cDC1 (*p* = 0.0007), and CD14^dim^ CD16^+^ monocytes (*p* = 0.0055) from older donors upregulated PD-L1 (Fig. 5c).

We also examined the eLNP-induced cytokine and chemokine profile of PBMCs. Empty LNP induced cytokine and chemokine profiles with elevated levels of expression after 24 h of stimulation specifically; IL-6 (young, *p* = 0.0142; old, *p* = 0.0076), IL-21 (young, *p* = 0.0020; old, *p* = 0.0030),IFNγ (young, *p* < 0.0001; old, *p* < 0.0001),TNF-a (young, *p* = 0.1069; old, *p* = 0.0420), CXCL13 (young, *p* = 0.0939; old, *p* = 0.0025), CCL7 (young, *p* = 0.0010; old, *p* = 0.0078). Overall, these data suggest that eLNP is immunostimulatory even in older adults which combined with antigen can lead to a sufficient vaccine response.

### eLNP stimulation results in impairment of phagocytosis in CD14^dim^CD16^+^ monocytes in older adults

**Fig. 6:**
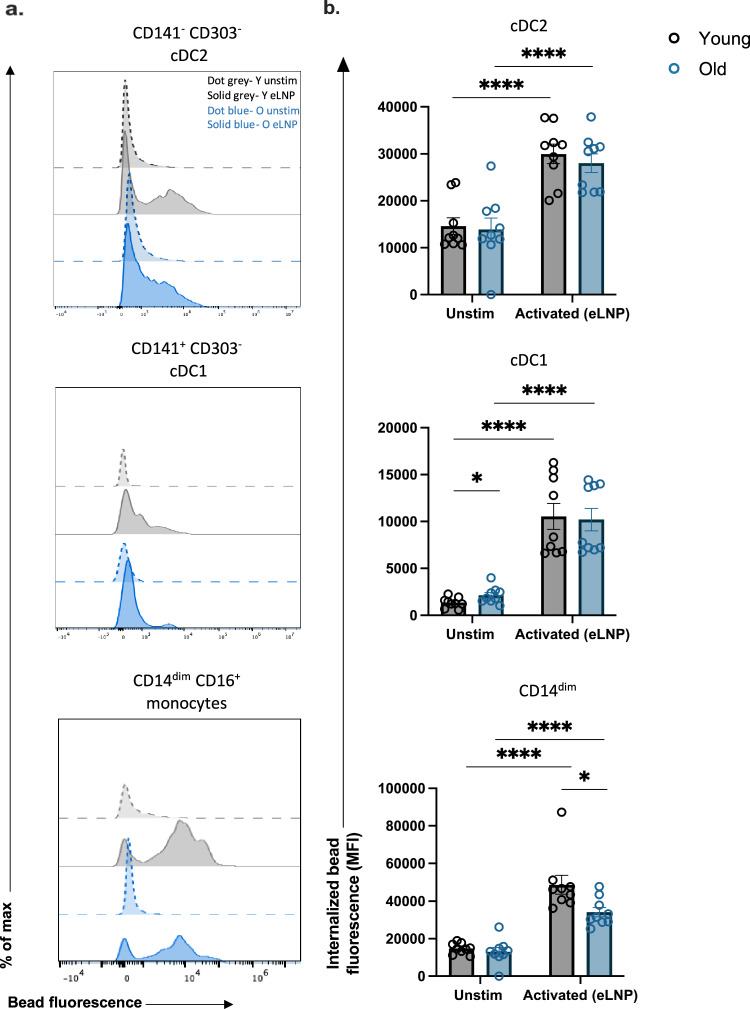
Phagocytosis is induced in response to eLNP stimulation. **a**, **b** Phagocytosis is induced in cDC2, cDC1, CD14^dim^ CD16^+^ monocytes. Phagocytosis measured using fluorescent beads and multiparametric flow cytometry. PBMCs were incubated overnight with stimulus and with beads for a further 3 h. Dotted grey line represents an example of a young donor unstimulated. Solid grey represents an example of a young donor stimulated with eLNP. Dotted blue line represents an example of an older donor unstimulated. Solid blue represents an example of an older donor stimulated with eLNP. Each individual circle represents one individual subject. *n* = 9, *P < 0.05, **P < 0.01, ***P < 0.001, and ****P < 0.0001 by non-parametric Mann Whitney T-test.

Upon stimulation with eLNP, all subtypes of monocytes and DCs showed increased phagocytosis in both young and older adults (Fig. 6a, b). Although eLNP enhanced the phagocytic ability of monocyte and DC subsets in all individuals, monocyte subtypes in aged individuals had reduced phagocytic activity (Fig. 6b).

In DC subsets, there was no significant difference in phagocytosis between young and older adults in response to stimuli, but phagocytosis was induced in cDC2 (young: eLNP, *p* = < 0.0001; old: eLNP, *p* = < 0.0001) and cDC1 (young: eLNP, *p* = < 0.0001; old: eLNP, *p* = < 0.0001). Empty LNP was capable of inducing phagocytosis in younger adults in CD14^dim^ CD16^+^ with eLNP (*p* = 0.0010). CD14^dim^ CD16^+^ from older adults had significant decrease in phagocytosis compared to younger adults when stimulated with eLNP (*p* = 0.0106). Of note, cDC2 showed significant decrease in phagocytic ability in older adults in LPS/IFN-γ levels (*p* = 0.0091), which predicts that cDC2’s role in T cell differentiation and GC reaction might also significantly impaired in older adults (Supplemental Fig. 5a). This impairment was easily rescued when induced with eLNP and had similar amount of phagocytic function throughout the DC subsets, which further underscores the ability of eLNP to enhance phagocytosis.

### eLNP mediates differential production of TGF-β production between younger and older adults

**Fig. 7:**
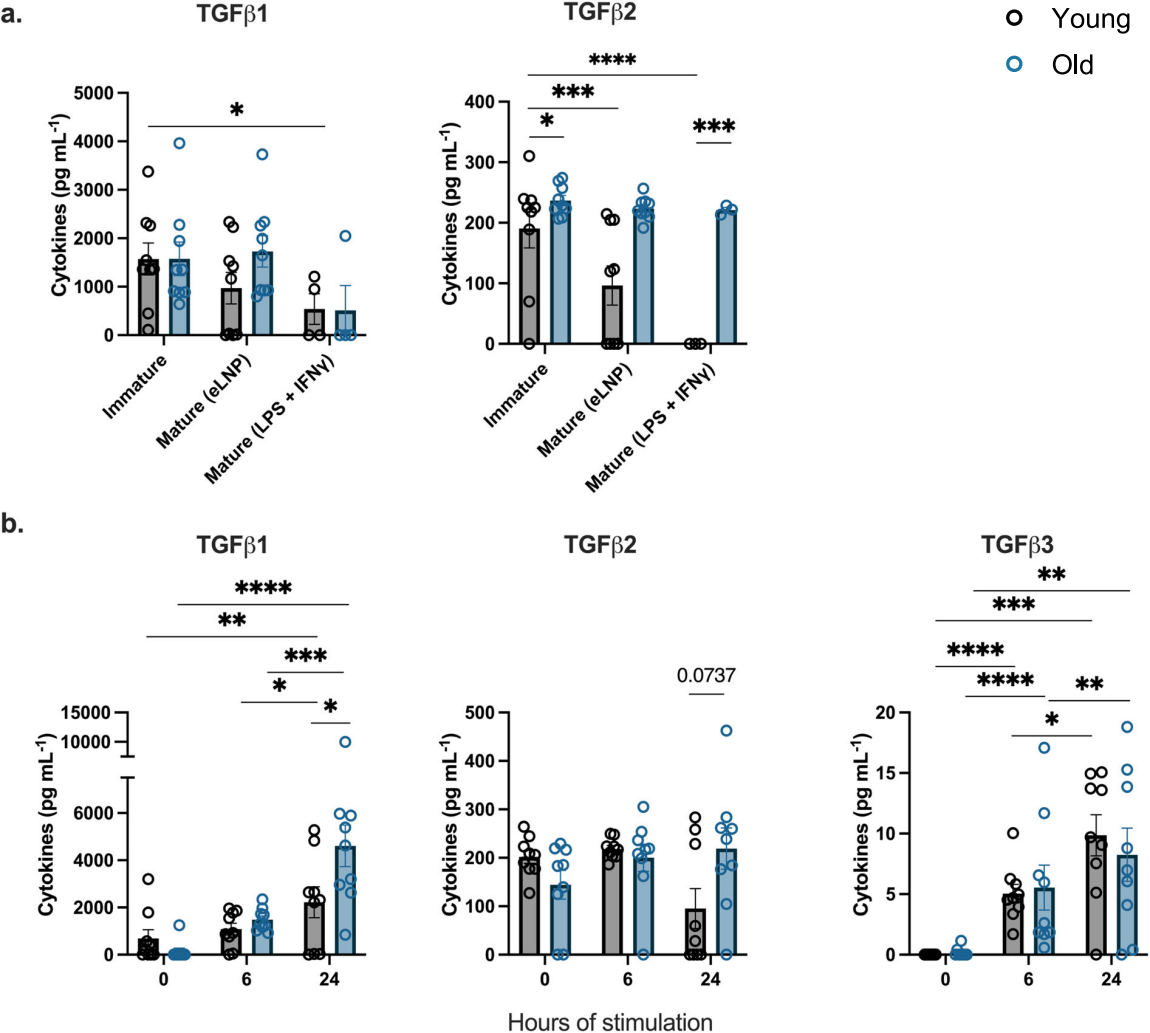
eLNP treatment results in differential TGF-β secretion. **a** TGFB secretion after 24 h eLNP stimulation compared to that in unstimulated cells of MDDCs. *n* = 9. Data were combined from at least two independent experiments. **b** Dynamics of TGF-B secretion from PBMCS at 0, 6, and 24 h after eLNP stimulation. *n* = 9 for each group. Data are from one independent experiment (a,b) Each individual circle represents one individual subject. One-way ANOVA followed by Tukey’s test was applied in **a**. Non-parametric Mann Whitney T-test was applied in **b**. *P < 0.05, **P < 0.01, ***P < 0.001, ****P < 0.0001.

To further understand the age differences following eLNP stimulation, TGF-β production by MDDCs and PBMCs from young and old participants was explored. For MDDCs, differences in TGF-β2 production were observed between young and old in immature cells (*p* = 0.0232) (Fig. 7a). However, following a 24 h treatment with eLNP, TGF-β2 production by MDDCs from young individuals was significantly lower than that found in older healthy adults (*p* = 0.0005) (Fig. 7a). Additionally, eLNP treatment led to a reduction in the production of all isoforms of TGF-β in aged individuals compared to levels detected from unstimulated MDDCs in the same aged individuals. In contrast, TGF-β production by MDDCs from young individuals was not found to be altered when compared to unstimulated MDDCs from the same young participants.

To gain a better understanding of the ability of eLNP treatment to initiate TGF-β production, PBMCs isolated from both healthy young and aged participants were treated with eLNP for either 6 or 24 h (Fig. 7b). Although no significant differences were initially observed between stimulated and unstimulated cells after 6 h in TGF-β1 and TGF-β2, TGF-β3 levels were significantly higher in both young (p *p* < 0.0001) and older participants (p *p* < 0.0001). TGF-β1 (young, *p* = 0.0034; old, *p* < 0.0001) and TGF-β3 (young, *p* = 0.0004; old, *p* = 0.0022) showed significant expression in both young and older adults after 24 h stimulation with eLNP. Importantly, other than TGF-β2 at 24 h stimulation (*p* = 0.0737), TGF-β1 production in young participants was reduced compared to old at 24 h (*p* = 0.0047) (Fig. 7b). Altogether, these data suggest that eLNP treatment is a potent inducer of TGF-β production in PBMCs while also suggesting that there are age-associated differences in the ability of eLNP to stimulate TGF-β production in young vs aged healthy adults. The ability of eLNP to induce TGF-β production and the differences observed between young and aged adults in the level of TGF-β that is induced are likely of importance, particularly in the use of eLNP during vaccination, due to the essential roles that TGF-β plays in the differentiation and function of T cells.

## Discussion

Aging is associated with increased morbidity and mortality to viral infections including SARS-CoV-2^35^. Of the multiple factors that contribute to this, impaired vaccine responsiveness and the creation of immunological memory are key contributors. To continue to identify vaccine platforms and adjuvants that strongly promote immune responses in older adults is critically important. Recently, modified nucleoside mRNA vaccines were approved to control the ongoing pandemic caused by SARS-CoV-2 (COVID-19). These vaccines utilize four-component lipid nanoparticles (LNPs) that have been shown to potently induce IL-1, IL-6 secretion, T_FH_ and GC B cell generation in humans^1,10,36–38^. As more mRNA-LNP vaccines are being designed for other infectious diseases, studies investigating the mechanism of immune modulation including the effect of LNP on innate immunity are critical to improve the efficacy of vaccines in older populations.

In this study, we expand upon the mechanism of ionizable eLNP formulation that Alameh et al. previously investigated. It was reported that the adjuvant properties of this LNP formulation are linked to the ionizable lipid component of the LNP and that there is an intrinsic ability of the eLNP to promote IL-6 secretion in mice and subsequent T_FH_ induction. We show that eLNP formulation induces the maturation and activation of DCs and monocytes as measured by the frequency of co-stimulatory surface receptors and the production of cytokines and chemokines (Fig. 1). We also show that there are age-specific differences in the maturation and activation in both MDDCs and subsets from PBMCs, namely cDC1, cDC2, and CD14^dim^ CD16^+^ monocytes (Fig. 5). This suggests the possibility that eLNP may mediate its effect on germinal center formation and T_FH_ function at least in part, by altering the maturation status of antigen- presenting cells (APCs)^1^. While Alameh et al show that the adjuvant effect of eLNP is maintained in MyD88-and MAVS-deficient mice, in human PBMCs, we show that eLNP treatment can induce the phosphorylation of IRF7 and TBK-1 (Fig. 2). The latter are key signaling molecules in antiviral responses, and their activation suggest that eLNP induce cytokine responses through toll like receptors (TLRs)-2, TLR3 and/or TLR4 or through other sensors that relay signaling via the TBK-1/IRF7 pathway. A recent study by Li *et al*, showed that immune responses to the Pfizer mRNA-LNP vaccine were not impaired in TLR-2, 4, 5 knockout mice. Immune responses were also not impaired in inflammasome (NLRP3), cGAS/STING, or RIPK3/GSDMD (danger associated molecular pattern (DAMP) sensors) knockout mice models suggesting that our finding that eLNP induce TKB-1/IRF7 pathway can be induced through unknown sensors, multiple convergent pathways (including IFNAR), and/or through other DAMP signaling molecules induced by LNP entry/or toxicity (cell death)^11^. TBK-1 induces innate antiviral type I IFNs but also plays a much broader role in antibody formation and autophagy^39,40^. In fact, it has also been demonstrated that TBK-1 signaling is, at least partially, responsible for mediating the adjuvant effect of DNA vaccines by differentially controlling DNA-activated innate immune signaling^41^. Of note, a recent study by Takanohashi, Alameh et al. 2022 looked at interferon stimulated genes (ISG) in response to eLNP transfected whole blood^42^. Upon 6 h eLNP transfection, this group did not see an elevated ISG score nor an induction of TBK-1 transcripts in healthy controls. This difference in results obtained could be due to several factors such as: 1) the use of whole blood compared to the use of isolated PBMCs and purified DCs in our study (cell populations of interest in our study are infrequent in whole blood); 2) loss of LNP dose in whole blood through the Red Blood Cells (RBCs) hitchhiking phenomena where nanoparticles adsorb on the nanoparticle surface; and 3) our study looked at phosphorylation of IRF7 and TBK-1 and protein levels of cytokine and chemokine and not on transcript expression.

Importantly, we have found that in response to eLNP treatment, cDC2, cDC1, and CD14^dim^ CD16^+^ monocytes from older donors have upregulated PD-L1 when compared to younger counterparts (Fig. 5c). PD-L1, through virtue of its function, regulates the germinal center response by limiting T_FH_ differentiation and function^43^. However, the upregulation of PD-L1 on cDCs is a critical role to protect against killing of cytotoxic T lymphocytes (CTLs) and errant PD-L1 expression might contribute to the decrease in immune responsiveness in older adults.

To investigate the mechanism of eLNP as a regulator of T_FH_ differentiation and innate immunity, we evaluated TGF-β production. TGF-β is a potent modulator of proliferation, differentiation, and function of all of lymphocytes, dendritic cells, and macrophages, essentially regulating both innate and antigen-specific immunity. In monocytes and DCs, TGF-β1 is mostly suppressive through inhibition of cell proliferation and reduction of reactive oxygen and nitrogen species^44^. TGF-β1 also acts as a chemotactic factor and can induce migration in monocytes and can suppress the type I IFN response in alveolar macrophages^45,46^. We show that TGF-β expression was significantly increased following eLNP stimulation in PBMCs from older individuals. In contrast, TGF-β expression in MDDCs was reduced in younger individuals therefore maintain the imbalance between old vs young upon eLNP stimulation (Fig. 7a). However, at 24 h stimulation with eLNP, PBMCs from older adults significantly increase production of TGF-β1 when compared with their younger counterparts. Increased TGF-β1 production by these cells might play a role in the overall decreased antiviral and vaccination response. Coupled with our finding that the addition of eLNP significantly increases the ability of cDC2 and cDC1 from younger and older donors to phagocytose, there is an evident role of eLNP in modulating vaccine responsiveness. In fact, cDC2 show a significant decrease in phagocytic ability when stimulated with LPS/IFN-γ in older donors (Supplemental Fig. 6). That age-specific difference is rescued when stimulated with eLNP, bringing levels of phagocytosis to that of younger donors indicating that eLNP can potentially be enhancing overall phagocytosis. TGF-β signals through STAT3:STAT4 to promote the differentiation of T_FH_ cells^19^ however the molecule also plays a role in the differentiation of T helper 17 (T_H_17) cells in inflammatory conditions in the presence of IL-6. We show an induction of IL-6 in PBMCs when stimulated with eLNP in both younger and older donors (Fig. 5) suggesting multiple roles of TGF-β under eLNP stimulation.

**Fig. 8:**
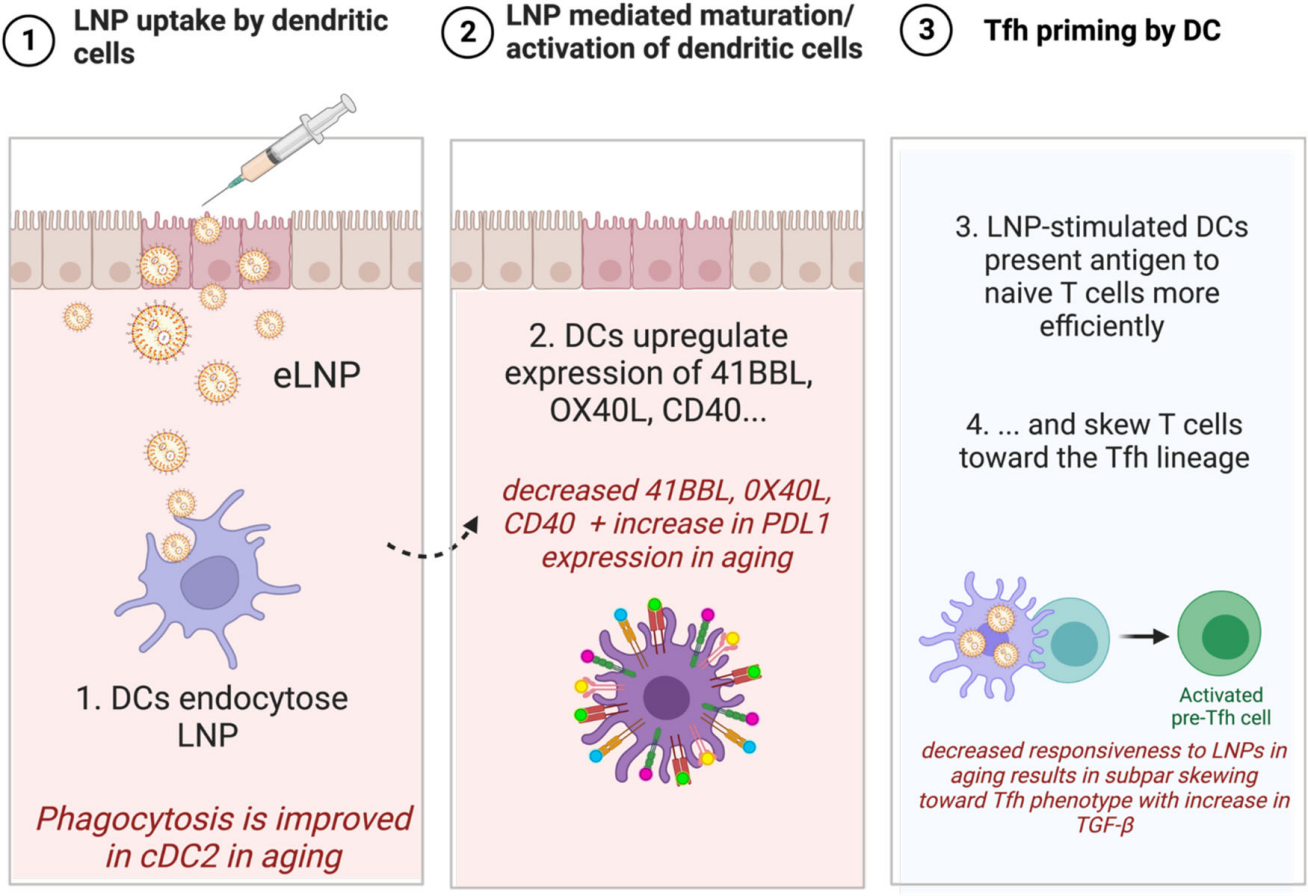
Lipid nanoparticles (LNP) can activate the innate immune response sans mRNA (eLNP). eLNP will upregulate co-stimulatory makers on the surface of monocytes, DCs, and PBMCs that are necessary for T cell activation and antigen-processing and presentation. eLNP will then induce a productive humoral and cellular response. In older adults, however, eLNP upregulates the same co-stimulatory but to a much lesser degree while also upregulating PD-L1, an inhibitory marker that was all but absent in younger adults. Addition of eLNP also induced TGF-B production in older adults. Taken together, our study shows a mechanism that accounts for the decreased protection due to vaccination in older adults.

A recent study showed that after vaccination with two doses of Pfizer–BioNTech mRNA vaccine (BNT162b2), pSTAT 1 and pSTAT3 was increased as well as the anti-inflammatory cytokine IL-10 further confirming a role for eLNP in initiating these responses^47^. We also show that in response to eLNP, IL-12 and IL-21 is secreted by MDDCs from older donors (Fig. 5b) and PBMCs from older and younger donors (Fig. 5d) suggesting a pro- T_FH_ inducing environment further indicating that eLNP is playing critical roles during vaccination^18^. We believe that our study provides important insight into the mechanism of action of eLNP such as those used in the Pfizer and Moderna COVID-19 vaccines (Fig. 8).

## Methods

### Human samples

Blood samples were obtained from healthy donors at Martin Memorial Health Systems (Florida). Consenting adults were screened using a questionnaire determining their demographic information, medication usage, and comorbidities. Participants were excluded with any acquired immunodeficiency or immunomodulating medications (such as steroids or chemotherapy), pregnancy, history of cancer, and history of cirrhosis or renal failure, or antibiotic use within 2 weeks of recruitment. Blood samples were taken from individuals aged 18–80 and were made into single cell PBMC suspensions and frozen in bovine serum albumin (Sigma) plus 10% DMSO (VWR) for cryopreservation in liquid nitrogen. The Institutional Review Boards at the relevant institutions approved all procedures, and all participants provided signed informed consent.

### Empty LNP generation

The LNP formulation used in this study is proprietary to Acuitas Therapeutics; the proprietary lipid and LNP composition are described in US patent US10,221,127. Briefly, an ethanolic lipid mixture of a proprietary ionizable lipid, DSPC, cholesterol, and polyethylene glycol-lipid was rapidly mixed with an acidic aqueous solution (PMID: 23799535^48^), dialyzed, concentrated, frozen and stored at −80 °C. Empty LNP were formulated at an equivalent mRNA concentration of 1 mg/ml and total lipid concentration of 30 mg/ml^11^. The mean hydrodynamic diameter of eLNP, measured by dynamic light scattering using a Zetasizer nano ZS (Malvern) was ~80 nm with a polydispersity index of 0.02–0.06.

### Determining optimal dose of eLNP

PBMCs were stimulated with eLNP in a twofold dilution to determine optimal dose. Optimal concentrations of eLNP were selected based on median production of IFN-γ and an 85% or more survival rate (Supplementary Fig. 7).

### Generation of human monocyte derived dendritic cells (MDDCs)

Previously cryopreserved human PBMCs from young (< 65 yrs old) and aged (> 65 yrs old) donors were thawed in RPMI 1640 (Corning) supplemented with 10% fetal bovine serum and 1% penicillin/streptomycin. PBMCs were enriched for CD14 + CD16 + monocytes via negative selection using EasySep**™** human monocyte enrichment kit without CD16 depletion (STEMCELL Technologies) according to the manufacturer’s protocol. Following enrichment, monocytes were counted and resuspended at a density of 2 × 10^6^ cells/mL in serum-free CellGenix® GMP dendritic cell medium (CellGenix) supplemented with 100 ng/mL of recombinant human GM-CSF (Gemini Bio-products) and 20 ng/mL of recombinant human IL-4 (Gemini Bio-Products). 1 mL of the resuspended cells was added in 24- well plates for a total of 1 × 10^6^ cells per well. Monocytes were differentiated for 48 h. prior to simulation.

### In vitro stimulation of MDDC

Monocytes were stimulated for 24 h with 0.5 µg/mL of LPS (Invivogen, Cat# TLRL-eblps) plus 40 ng/mL of IFN-γ (Invivogen, Cat#300-134 P) or 0.78 µg of empty lipid nanoparticles (eLNP) in 1 mL of GM-CSF and IL-4 supplemented medium. Unstimulated control cells were maintained in GM-CSF and IL-4 supplemented medium for 24 h. Following stimulation, dendritic cells were harvested and analyzed via flow cytometry and supernatants were collected after stimulation and frozen at −80 °C

### Flow cytometric analysis of human MDDCs

Briefly, after stimulation, harvested MDDCs were washed twice with fluorescence-activated cell sorting (FACS) buffer (PBS containing 2% FBS), surface stained using antibodies for 30 minutes in 100 ul of FACS buffer. LIVE/DEAD Fixable Dead Cell Stain (Life Technologies, Cat: L34957) was used to gate on live cells (see Supplemental Table 2). Samples were acquired on a BD^TM^ LTR Fortessa (BD Biosciences), and analysis was conducted using FlowJo software (version 10). When gating, doublet cells were excluded and MDDCs were gated on live CD3^-^ CD19^-^ CD56^-^ CD11c^+^ cells.

### In vitro stimulation of monocyte and DC subsets

PBMCs from healthy young and older donors were plated at a volume of 1.0 × 10^6^ cells per well in a round 96-well plate in a volume of 100 ul of complete RPMI medium (RPMI 1640 with L-glutamine [Corning Cellgro, Manassas, VA] supplemented with 10% FBS and 13 [50 U] penicillin-streptomycin [Invitrogen, Carlsbad, CA]). For experiments involving LPS/ IFNg stimulation, PBMCs were stimulated for 6 or 24 h with 0.5 ug/mL LPS (Invivogen, Cat# TLRL-eblps) plus 40 ng/mL IFNg (Invivogen, Cat#300-134 P). For experiments involving empty LNP, PBMCs were stimulated for 6 or 24 h with 0.78 ug/mL eLNP. Following stimulation, PBMCs were analyzed via flow cytometry and supernatants were collected after stimulation and frozen at −80 °C

### In vitro stimulation of isolated monocytes

Monocytes were isolated from PBMCs with the StemCell Technology monocyte negative selection kit (Cat# 19359). Isolated monocytes were stimulated for 24 h with 0.5 µg/mL of LPS (Invivogen, Cat# TLRL-eblps) plus 40 ng/mL of IFN-γ (Invivogen, Cat#300-134P) or 0.78 µg of empty lipid nanoparticles (eLNP) in 1 mL of GM-CSF and IL-4 supplemented medium. Unstimulated control cells were maintained in GM-CSF and IL-4 supplemented medium for 24 h. Following stimulation, dendritic cells were harvested and analyzed via flow cytometry and supernatants were collected after stimulation and frozen at −80 °C

### Phosflow cytometry analysis of monocyte and DC subsets and costimulatory markers

The stimulated PBMCs from adult or older adult donors were prepared and incubated with fluorochrome-conjugated antibodies for flow cytometry. Briefly, after stimulation, cells were washed twice with fluorescence-activated cell sorting (FACS) buffer (PBS containing 2% FBS), surface stained using antibodies for 30 min in 100 ul of FACS buffer, permeabilized using 300 µl of cold BD phosflow buffer III (BD Biosciences) according to manufacturer’s instructions, intracellular phosphoprotein stained using antibodies against intracellular phosphorylated IRF7(pS477/ pS479, BD Biosciences), pTBK-1 (BD Biosciences, or STING (BD Biosciences in 50 ul FACS buffer for 1 h, and then fixed using 2% PFA for 15 min at 37 °C. LIVE/DEAD Fixable Dead Cell Stain (Life Technologies, Cat: L34957) was used to gate on live cells. Samples were acquired on a BD^TM^ LTR Fortessa (BD Biosciences), and analysis was conducted using FlowJo software (version 10). Cells were phenotyped as follows: cDC2 were Lineage ^−^ (CD19^−^ CD3^−^ CD56^−^CD20−) HLA-DR+ CD11c+ CD1c+ CD141− CD303−, cDC1 were Lineage^−^ HLA-DR+ CD11c− CD1c− CD141+ CD303^−^, pDC were Lineage^−^ HLA-DR+ CD11c− CD1c− CD141− CD303+, Classical monocytes were Lineage^−^ HLA-DR+ CD14+ CD16−, Intermediate monocytes were Lineage^−^ HLA-DR^+^ CD14^+^ CD16^+^, and non-classical monocytes were Lineage^−^ HLA-DR^+^ CD14^dim^ CD16^+^ (see Supplementary Table 3).

### In vitro phagocytosis assay

PBMCs from healthy young and older donors were plated at a volume of 1.0 × 10^6^ cells per well in a round 96-well plate in a volume of 100 µl of complete RPMI medium at 37 °C and at 4 °C as a negative control. PBMCs were then stimulated as stated above for 24 h. After a 24 h stimulation period, cells were washed twice with FACS buffer to remove agonist and incubated with 0.04 µm fluorescent microspheres (Invitrogen, Cat: F8794) for 3 h. The cells were then washed twice with FACS buffer to remove any beads from the outside of the cell and prepared for flow cytometry.

### Phagocytic flow cytometry analysis of monocyte and DC subsets

The PBMCs from adult or older adult donors were prepared and incubated with fluorochrome-conjugated antibodies for flow cytometry. Briefly, after stimulation, cells were washed twice with FACS buffer, surface stained using antibodies for 30 min in 100 µl of FACS buffer, and then fixed using 2% PFA for 15 min at 37 °C. Cells were phenotyped as stated above.

### Cytokine and chemokine analysis

Supernatants collected from PBMCs during stimulation were analyzed for chemokine/cytokine levels using the human immune monitoring 65-Plex ProcartaPlex™ Panel (Invitrogen™). This kit was used to determine the levels of 65 cytokines, chemokines, growth factors, and soluble receptors produced by MDDCs, 24 h after stimulations, and PBMCs 6 and 24 h after stimulations. The following human chemokine/cytokine premixed panel was used according to the manufacturer’s protocol: G-CSF (CSF-3), GM-CSF, IFN alpha, IFN-g, IL-1a, IL-1b, IL-2, IL-3, IL-4, IL-5, IL-6, IL-7, IL-8 (CXCL8), IL-9, IL-10, IL-12p70, IL-13, IL-15, IL-16, IL-17A (CTLA-8), IL-18, IL-20, IL-21, IL-22, IL-23, IL-27, IL-31, LIF, M-CSF, MIF, TNF-a, TNF-b, TSLP, BLC (CXCL13), ENA-78 (CXCL5), Eotaxin (CCL11), Eotaxin-2 (CCL24), Eotaxin-3 (CCL26), Fractalkine (CX3CL1), Gro-alpha (CXCL1), IP-10 (CXCL10), I-TAC (CXCL11), MCP-1 (CCL2), MCP-2 (CCL8), MCP-3 (CCL7), MDC (CCL22), MIG (CXCL9), MIP-1a (CCL3), MIP-1b (CCL4), MIP-3a (CCL20), SDF-1a (CXCL12), FGF-2, HGF, MMP-1, NGF-b, SCF, VEGF-A, APRIL, BAFF, CD30, CD40L (CD154), IL-2R (CD25), TNF-RII, TRAIL (CD253), TWEAK. Data was acquired on a Luminex™ FLEXMAP 3D™ System using bead regions defined in the protocol and analyzed using Belysa Curve Fitting Software (Sigma Aldrich). Standard curves were generated, and sample concentrations were calculated in pg/mL.

### Statistics and Reproducibility

All flow cytometry, Luminex, and confocal data were analyzed using GraphPad Prism v9. Where appropriate, stimulations were subtracted from their background controls, i.e., LPS/IFNγ stimulated cells were subtracted from unstimulated, RIG-I agonist was subtracted from LyoVec only control, and G10 STING agonist was subtracted from a DMSO control. Unpaired, non-parametric Mann-Whitney test was used when comparing two groups. The Paired multiple t-test and non-parametric one-way ANOVA (Friedman) test was used when comparing more than two groups to each other. (*p < 0.05, **p < 0.01, ***p < 0.001, ****p < 0.0001).

### Reporting summary

Further information on research design is available in the Nature Portfolio Reporting Summary linked to this article.

## Supplementary information

Supplementary Information

Description of Additional Supplementary Files

Supplementary Data 1

Reporting Summary

## Data Availability

The datasets generated during and/or analysed during the current study are available as a supplementary data 1 file.

## References

[1] Alameh, M.-G. et al. Lipid nanoparticles enhance the efficacy of mRNA and protein subunit vaccines by inducing robust T follicular helper cell and humoral responses. *Immunity* **54**, 2877–2892.e2877 (2021). 34852217 10.1016/j.immuni.2021.11.001 PMC8566475

[2] Pardi, N. et al. Nucleoside-modified mRNA immunization elicits influenza virus hemagglutinin stalk-specific antibodies. *Nat. Commun.* **9**, 3361 (2018). 30135514 10.1038/s41467-018-05482-0 PMC6105651

[3] Swaminathan, G. et al. A Tetravalent sub-unit dengue vaccine formulated with ionizable cationic lipid nanoparticle induces significant immune responses in rodents and non-human primates. *Sci. Rep.* **6**, 34215 (2016). 27703172 10.1038/srep34215 PMC5050434

[4] Shirai, S. et al. Lipid nanoparticle acts as a potential adjuvant for influenza split vaccine without inducing inflammatory responses. *Vaccines* **8**, 433 (2020). 32756368 10.3390/vaccines8030433 PMC7565178

[5] Awasthi, S. et al. Antibody responses to crucial functional epitopes as a novel approach to assess immunogenicity of vaccine adjuvants. *Vaccine* **37**, 3770–3778 (2019). 31153687 10.1016/j.vaccine.2019.05.068 PMC6579625

[6] Alameh, M-G., Weissman, D., Pardi, N. Messenger RNA-Based Vaccines Against Infectious Diseases.). Springer Berlin Heidelberg. 10.1007/82_2020_202 32300916

[7] Barbier, A. J., Jiang, A. Y., Zhang, P., Wooster, R. & Anderson, D. G. The clinical progress of mRNA vaccines and immunotherapies. *Nat. Biotechnol.* **40**, 840–854 (2022). 35534554 10.1038/s41587-022-01294-2

[8] Ndeupen, S. et al. The mRNA-LNP platform’s lipid nanoparticle component used in preclinical vaccine studies is highly inflammatory. *iScience* **24**, 103479 (2021). 34841223 10.1016/j.isci.2021.103479 PMC8604799

[9] Swaminathan, G. et al. Activation of the TLR2-MyD88 pathway is required for in-vivo efficacy of Lipid Nanoparticle based vaccine formulation. *J. Immunol.* **198**, 79.72 (2017).

[10] Tahtinen, S. et al. IL-1 and IL-1ra are key regulators of the inflammatory response to RNA vaccines. *Nat. Immunol.* **23**, 532–542 (2022). 35332327 10.1038/s41590-022-01160-y

[11] Li, C. et al. Mechanisms of innate and adaptive immunity to the Pfizer-BioNTech BNT162b2 vaccine. *Nat. Immunol.* **23**, 543–555 (2022). 35288714 10.1038/s41590-022-01163-9 PMC8989677

[12] Derhovanessian, E. & Pawelec, G. Vaccination in the elderly. *Microb. Biotechnol.* **5**, 226–232 (2012). 21880118 10.1111/j.1751-7915.2011.00283.x PMC3815782

[13] CDC. Severe Outcomes Among Patients with Coronavirus Disease 2019 (COVID-19) — United States, February 12–March 16, 2020. (2020). 10.15585/mmwr.mm6912e2 PMC7725513 32214079

[14] Wu, Y., Goplen, N. P. & Sun, J. Aging and respiratory viral infection: from acute morbidity to chronic sequelae. *Cell Biosci.* **11**, 112 (2021). 34158111 10.1186/s13578-021-00624-2 PMC8218285

[15] Collier, D. A. et al. Age-related immune response heterogeneity to SARS-CoV-2 vaccine BNT162b2. *Nature* **596**, 417–422 (2021). 34192737 10.1038/s41586-021-03739-1 PMC8373615

[16] Parry, H. et al. mRNA vaccination in people over 80 years of age induces strong humoral immune responses against SARS-CoV-2 with cross neutralization of P.1 Brazilian variant. *eLife* **10**, e69375 (2021). 34586068 10.7554/eLife.69375 PMC8500710

[17] Rydyznski Moderbacher, C. et al. Antigen-Specific Adaptive Immunity to SARS-CoV-2 in Acute COVID-19 and Associations with Age and Disease Severity. *Cell* **183**, 996–1012.e1019 (2020). 33010815 10.1016/j.cell.2020.09.038 PMC7494270

[18] Schmitt, N. et al. Human Dendritic Cells Induce the Differentiation of Interleukin-21-Producing T Follicular Helper-like Cells through Interleukin-12. *Immunity* **31**, 158–169 (2009). 19592276 10.1016/j.immuni.2009.04.016 PMC2731623

[19] Schmitt, N. et al. The cytokine TGF-β co-opts signaling via STAT3-STAT4 to promote the differentiation of human TFH cells. *Nat. Immunol.* **15**, 856–865 (2014). 25064073 10.1038/ni.2947 PMC4183221

[20] Coccia, M. et al. Cellular and molecular synergy in AS01-adjuvanted vaccines results in an early IFNγ response promoting vaccine immunogenicity. *npj Vaccines* **2**, 25 (2017). 29263880 10.1038/s41541-017-0027-3 PMC5627273

[21] Connors, J. et al. Aging alters antiviral signaling pathways resulting in functional impairment in innate immunity in response to pattern recognition receptor agonists. *GeroScience* **44**, 2555–2572 (2022). 35849213 10.1007/s11357-022-00612-5 PMC9289086

[22] Palucka, K. & Banchereau, J. Human dendritic cell subsets in vaccination. *Curr. Opin. Immunol.* **25**, 396–402 (2013). 23725656 10.1016/j.coi.2013.05.001 PMC3711217

[23] Reuter, A. et al. Criteria for dendritic cell receptor selection for efficient antibody-targeted vaccination. *J. Immunol.* **194**, 2696 (2015). 25653426 10.4049/jimmunol.1402535

[24] Pooley, J. L., Heath, W. R. & Shortman, K. Cutting edge: intravenous soluble antigen is presented to CD4 T cells by CD8- dendritic cells, but cross-presented to CD8 T cells by CD8+ dendritic cells. *J. Immunol.* **166**, 5327 (2001). 11313367 10.4049/jimmunol.166.9.5327

[25] Calabro, S. et al. Differential Intrasplenic Migration of Dendritic Cell Subsets Tailors Adaptive Immunity. *Cell Rep.* **16**, 2472–2485 (2016). 27545885 10.1016/j.celrep.2016.07.076 PMC6323650

[26] Dudziak, D. et al. Differential Antigen Processing by Dendritic Cell Subsets in Vivo. *Science* **315**, 107–111 (2007). 17204652 10.1126/science.1136080

[27] Guilliams, M. et al. Unsupervised High-Dimensional Analysis Aligns Dendritic Cells across Tissues and Species. *Immunity* **45**, 669–684 (2016). 27637149 10.1016/j.immuni.2016.08.015 PMC5040826

[28] Cros, J. et al. Human CD14 Monocytes Patrol and Sense Nucleic Acids and Viruses via TLR7 and TLR8 Receptors. *Immunity* **33**, 375–386 (2010). 20832340 10.1016/j.immuni.2010.08.012 PMC3063338

[29] Ziegler-Heitbrock, L. The CD14+ CD16+ blood monocytes: their role in infection and inflammation. *J. Leukoc. Biol.* **81**, 584–592 (2007). 17135573 10.1189/jlb.0806510

[30] Kawai, T. et al. Interferon-α induction through Toll-like receptors involves a direct interaction of IRF7 with MyD88 and TRAF6. *Nat. Immunol.* **5**, 1061 (2004). 15361868 10.1038/ni1118

[31] Liu, G. et al. Nuclear-resident RIG-I senses viral replication inducing antiviral immunity. *Nat. Commun.* **9**, 3199 (2018). 30097581 10.1038/s41467-018-05745-w PMC6086882

[32] Oh, S. A. & Li, M. O. TGF-β: guardian of T cell function. *J. Immunol.* **191**, 3973–3979 (2013). 24098055 10.4049/jimmunol.1301843 PMC3856438

[33] Tahiliani, V., Hutchinson, T. E., Abboud, G., Croft, M. & Salek-Ardakani, S. OX40 Cooperates with ICOS To Amplify Follicular Th Cell Development and Germinal Center Reactions during Infection. *J. Immunol.* **198**, 218 (2017). 27895177 10.4049/jimmunol.1601356 PMC5173420

[34] Wolfl, M. et al. Activation-induced expression of CD137 permits detection, isolation, and expansion of the full repertoire of CD8+ T cells responding to antigen without requiring knowledge of epitope specificities. *Blood* **110**, 201–210 (2007). 17371945 10.1182/blood-2006-11-056168 PMC1896114

[35] Yang, W. et al. Estimating the infection-fatality risk of SARS-CoV-2 in New York City during the spring 2020 pandemic wave: a model-based analysis. *Lancet Infect. Dis.* **21**, 203–212 (2021). 33091374 10.1016/S1473-3099(20)30769-6 PMC7572090

[36] Lederer, K. et al. Germinal center responses to SARS-CoV-2 mRNA vaccines in healthy and immunocompromised individuals. *Cell* **185**, 1008–1024.e15 (2022). 35202565 10.1016/j.cell.2022.01.027 PMC8808747

[37] Mudd, P. A. et al. SARS-CoV-2 mRNA vaccination elicits a robust and persistent T follicular helper cell response in humans. *Cell* **185**, 603–613.e615 (2022). 35026152 10.1016/j.cell.2021.12.026 PMC8695127

[38] Turner, J. S. et al. SARS-CoV-2 mRNA vaccines induce persistent human germinal centre responses. *Nature* **596**, 109–113 (2021). 34182569 10.1038/s41586-021-03738-2 PMC8935394

[39] Clément, J.-F., Meloche, S. & Servant, M. J. The IKK-related kinases: from innate immunity to oncogenesis. *Cell Res.* **18**, 889–899 (2008). 19160540 10.1038/cr.2008.273

[40] Stolz, A., Ernst, A. & Dikic, I. Cargo recognition and trafficking in selective autophagy. *Nat. Cell Biol.* **16**, 495–501 (2014). 24875736 10.1038/ncb2979

[41] Ishii, K. J. et al. TANK-binding kinase-1 delineates innate and adaptive immune responses to DNA vaccines. *Nature* **451**, 725–729 (2008). 18256672 10.1038/nature06537

[42] Takanohashi, A. et al. mRNA-based vaccines against SARS-CoV-2 do not stimulate interferon stimulatory gene expression in individuals affected by Aicardi Goutières Syndrome. *bioRxiv*, 10.1101/2022.05.18.492546 (2022).

[43] Krishnaswamy Jayendra, K. et al. Migratory CD11b+ conventional dendritic cells induce T follicular helper cell–dependent antibody responses. *Sci. Immunol.* **2**, eaam9169 (2017). 29196450 10.1126/sciimmunol.aam9169 PMC7847246

[44] Kubiczkova, L., Sedlarikova, L., Hajek, R. & Sevcikova, S. TGF-β – an excellent servant but a bad master. *J. Transl. Med.* **10**, 183 (2012). 22943793 10.1186/1479-5876-10-183 PMC3494542

[45] Wahl, S. M. et al. Transforming growth factor type beta induces monocyte chemotaxis and growth factor production. *Proc. Natl Acad. Sci.* **84**, 5788–5792 (1987). 2886992 10.1073/pnas.84.16.5788 PMC298948

[46] Grunwell, J. R. et al. TGF-β1 Suppresses the Type I IFN response and induces mitochondrial dysfunction in alveolar macrophages. *J. Immunol.* **200**, 2115 (2018). 29427413 10.4049/jimmunol.1701325 PMC5928790

[47] Arunachalam, P. S. et al. Systems vaccinology of the BNT162b2 mRNA vaccine in humans. *Nature* **596**, 410–416 (2021). 34252919 10.1038/s41586-021-03791-x PMC8761119

[48] Maier, M. A. et al. Biodegradable lipids enabling rapidly eliminated lipid nanoparticles for systemic delivery of RNAi therapeutics. *Mol. Ther.* **21**, 1570–1578 (2013). 23799535 10.1038/mt.2013.124 PMC3734658

