# Author Correction: Lipid nanoparticles (LNP) induce activation and maturation of antigen presenting cells in young and aged individuals

**DOI:** 10.1038/s42003-025-07603-0

**Published:** 2025-02-22

**Authors:** Jennifer Connors, David Joyner, Nathan J. Mege, Gina M. Cusimano, Matthew R. Bell, Jennifer Marcy, Bhavani Taramangalam, Kenneth M. Kim, Paulo J. C. Lin, Ying K. Tam, Drew Weissman, Michele A. Kutzler, Mohamad-Gabriel Alameh, Elias K. Haddad

**Affiliations:** 1 Drexel University College of Medicine, Department of Microbiology and Immunology, Philadelphia, PA USA; 2 Drexel University College of Medicine, Department of Medicine, Philadelphia, PA USA; 3 Drexel University College of Medicine, Department of Molecular and Cellular Biology, Philadelphia, PA USA; 4 Tower Health, Reading Hospital, Reading, PA USA; 5 Acuitas Therapeutics, Vancouver, Canada; 6 University of Pennsylvania, Perelman School of Medicine, Philadelphia, PA USA; 7 University of Pennsylvania, Penn Institute for RNA Innovation, Philadelphia, PA USA

**Keywords:** Innate immune cells, Adjuvants

Correction to: *Communications Biology* 10.1038/s42003-023-04555-1, published online 17 February 2023

In the version of the article initially published, the “24 h” data points in Fig. 2b were duplicates of the “6 h” data and have now been corrected in the HTML and PDF versions of the article.

