# Supplementary material for: Lipid nanoparticles (LNP) induce activation and maturation of antigen presenting cells in young and aged individuals
Source: Commun Biol. 2023 Feb 17;6:188. doi: 10.1038/s42003-023-04555-1 (PMC9936473; doi:10.1038/s42003-023-04555-1)
Supplement: Supplementary file 1 — Supplementary Information [file 42003_2023_4555_MOESM1_ESM.pdf]

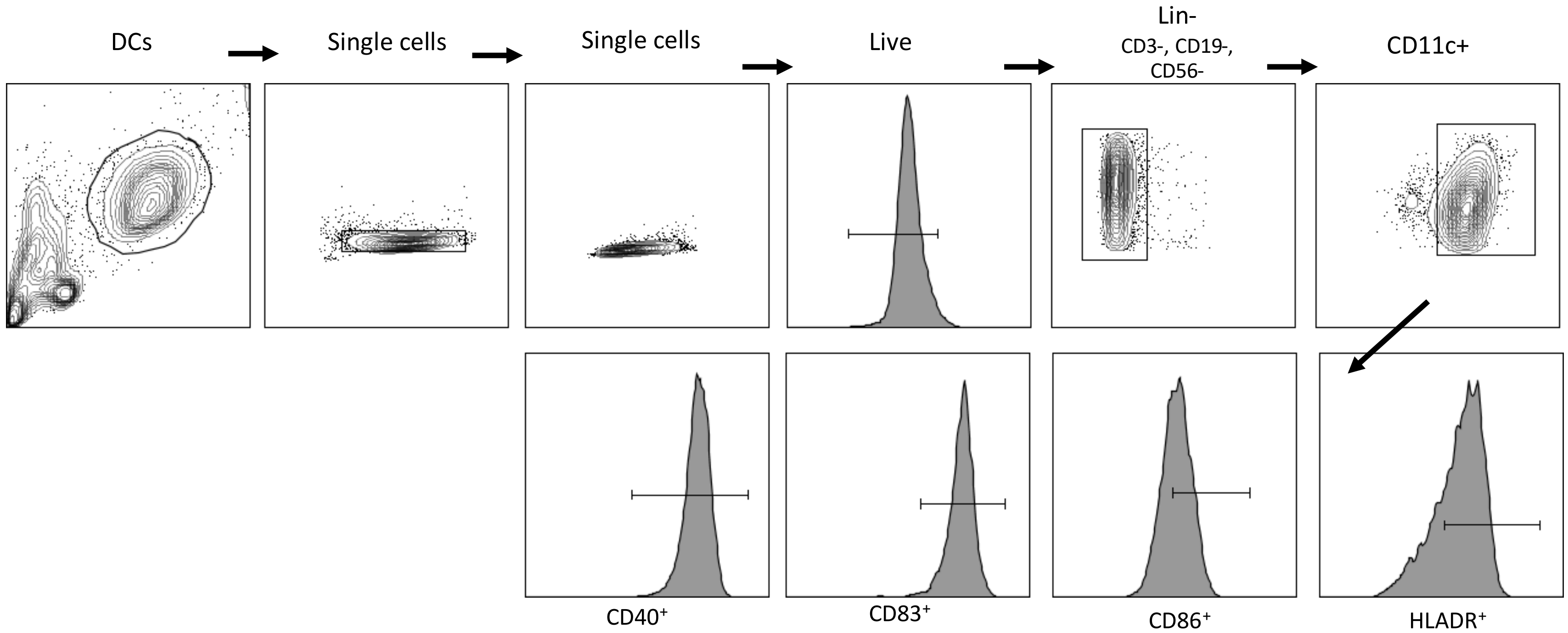

Supplemental Fig 1. MDDC gating strategy (a) Representative conventional flow cytometry plots from healthy adult monocyte derived dendritic cells (MDDC) showing the hierarchical phenotype staining from P1 to CD40+, CD83+, CD86+, or HLA-DR+.

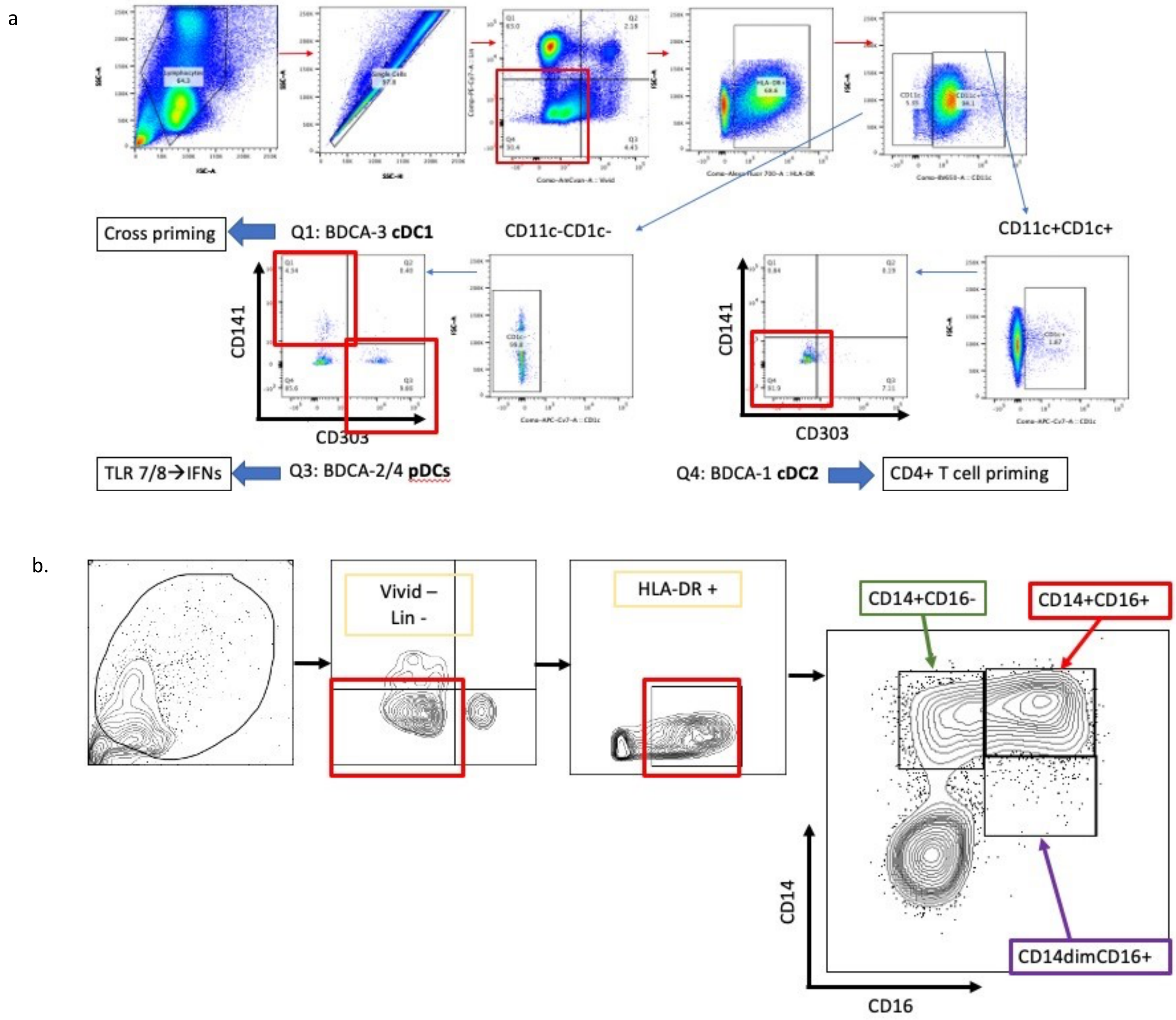

Supplemental fig 2: PBMC gating strategy. (a) Representative conventional flow cytometry plots from healthy adult PBMCs showing the hierarchical phenotype staining from P1 to subsets of dendritic cells, (b) monocytes.

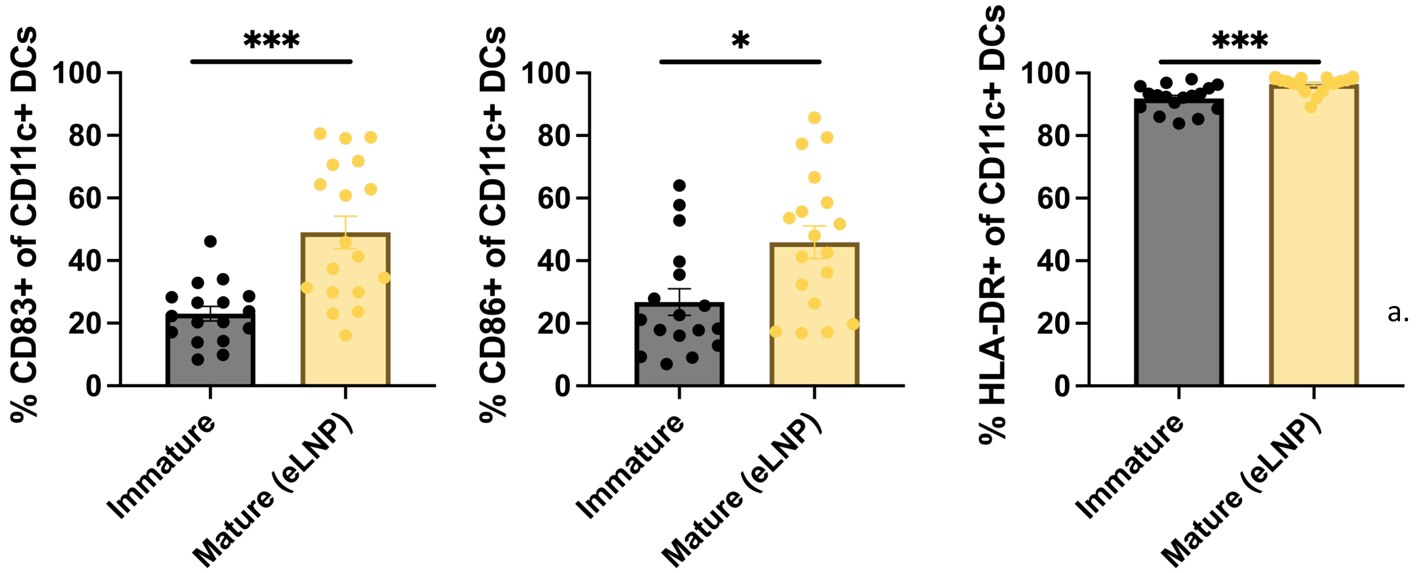

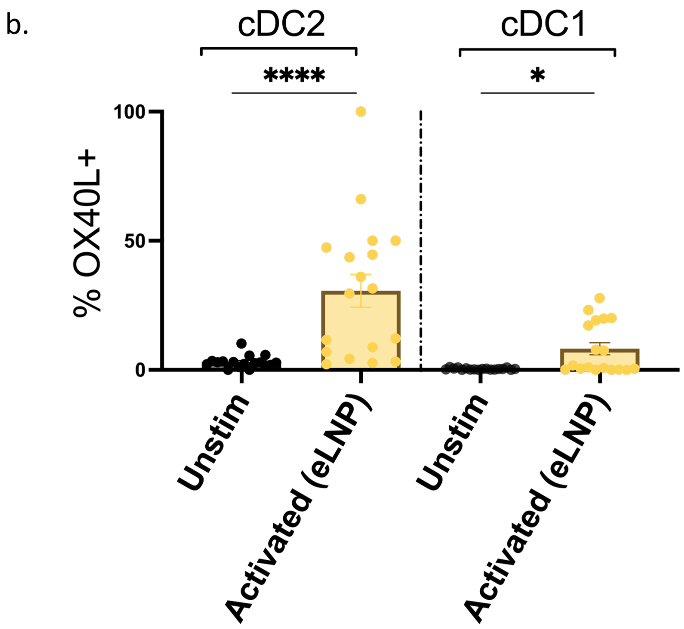

Supplemental Fig 3: eLNP treatment promotes robust maturation and antigen presentation activation

a Activation status of monocyte-derived DCs as measured by CD83, CD86, and HLA-DR expression after 24 hours of eLNP stimulation (yellow). *n* = 18 **b** Innate cell activation after 24-hour eLNP stimulation (yellow) compared to that in unstimulated (media) cells (grey) of PBMCs as measured by OX40L upregulation. *n* = 18 for each group. Data are from one independent experiment. Each individual circle represents one individual subject. Non-parametric Mann Whitney T-test was applied in a,b *$P < 0.05$, **$P < 0.01$, ***$P < 0.001$, ****$P < 0.0001$.

a.

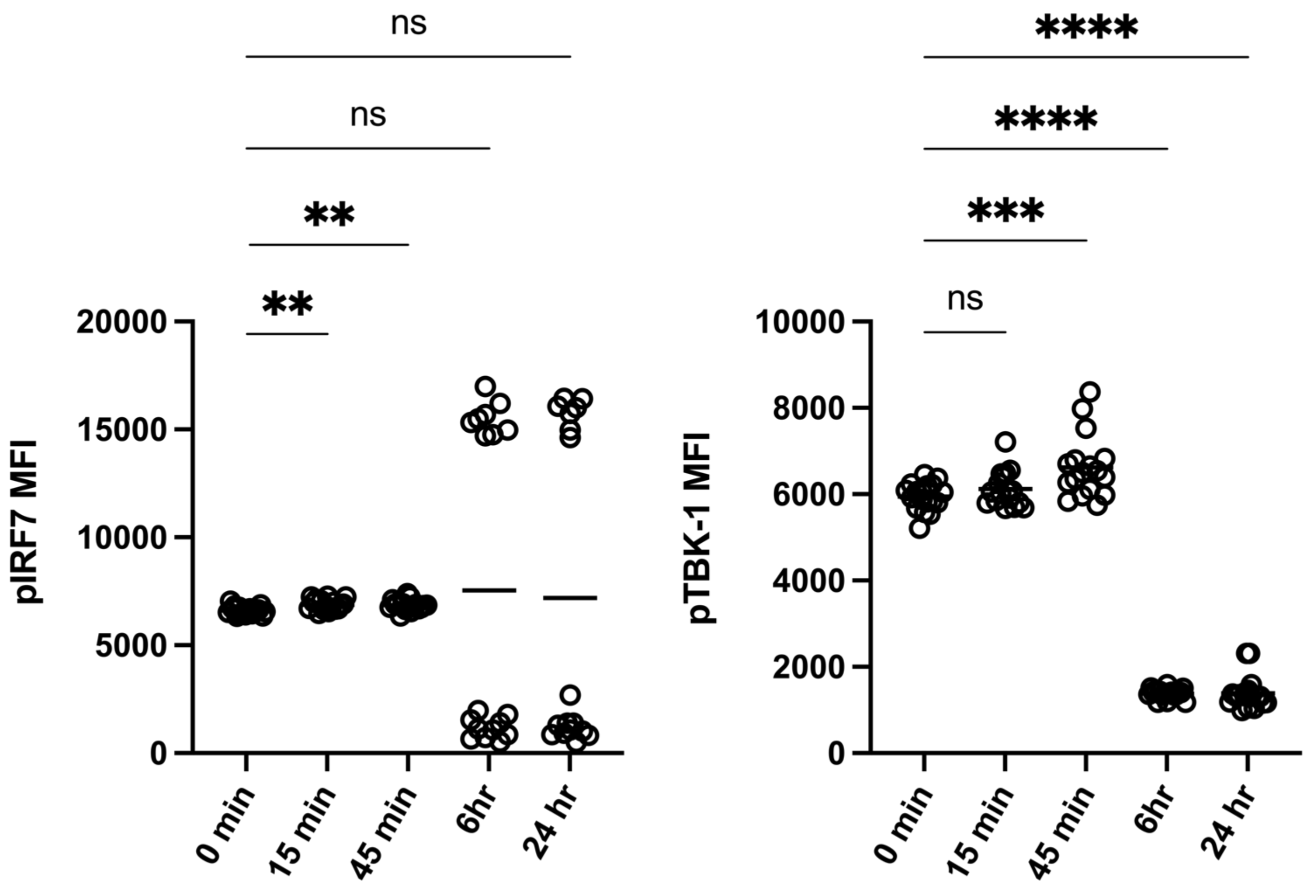

Supplemental 4: IRF7 and TBK-1 activation not significantly different in CD14dim CD16+ monocytes

Human PBMCs from healthy donors were either stimulated with 0.78ug/mL of eLNP for 15 min, 45 min, 6, or 24 hours. For unstimulated and control conditions refer to figure 1. Cells were permeabilized, fixed, and stained for **a** phosphorylated interferon response factor 7 (IRF7) transcription factor or **b** phosphorylated TBK-1. After gating on monocyte and DC subsets, the geometric mean intensity (MFI) was measured using phosflow cytometry. Each individual circle represents one individual subject. N=9*P < 0.05, **P < 0.01, ***P < 0.001, and ****P < 0.0001 by one-way ANOVA per time point.

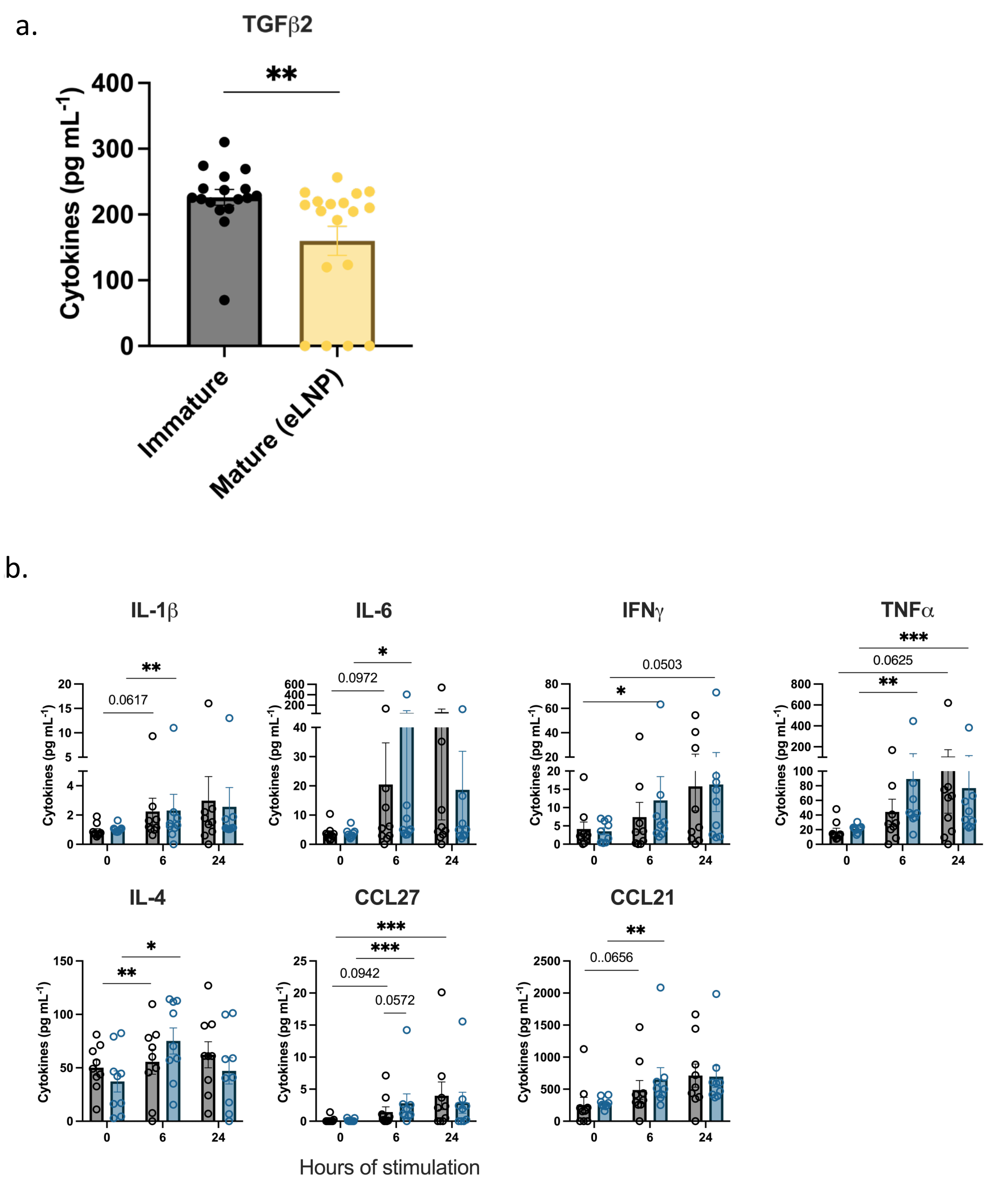

Supplemental Fig 5: MDDC have decreased TGFB production and monocytes show no significant differences in response to eLNP stimulation

a Monocyte-derived dendritic cells (MDDCs) differentiated *in vitro* from monocytes of healthy human PBMCs from donors were treated with 15 µg/mL (total lipids, or~7.5 µg/mL ionizable lipid) eLNP for 24 h and compared to unstimulated cells (just media) as controls. TGF-B isoforms were measured by Luminex. *n* = 18 for each group. **b** Monocytes isolated from healthy human PBMCs were treated 15 µg/mL (total lipids, or~7.5 µg/mL ionizable lipid) eLNP for 24 h and compared to unstimulated cells (just media) as controls. Cytokine and chemokine secretion was measured by Luminex. Nine samples per group were measured, except 24h LPS where only three samples per group were analyzed. Each individual circle represents one individual subject. *P < 0.05, **P < 0.01, ***P < 0.001, and ****P < 0.0001 by one-way ANOVA or non-parametric Mann Whitney T-test

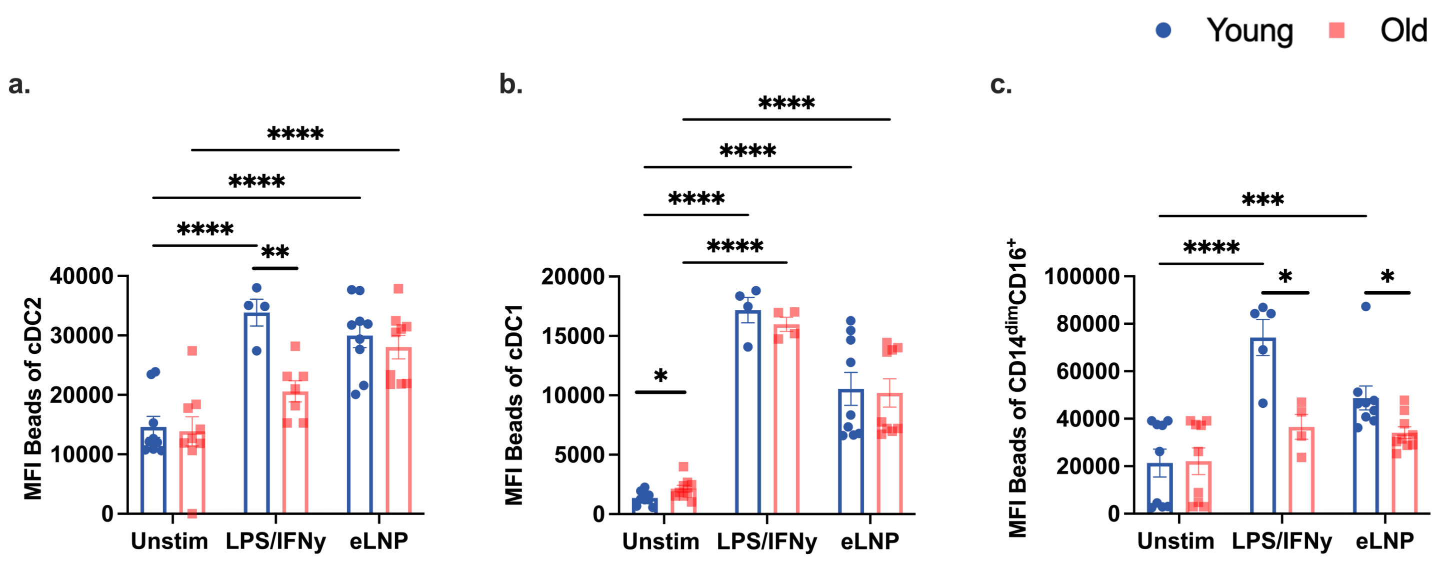

Supplemental figure 6: Phagocytosis is increased in response to eLNP stimulation but is impaired in CD14^{dim} CD16^{+} monocytes in older adults.

A-F. Phagocytosis is less efficient in (a)cDC2, (b)cDC1, (c) CD14^{dim} CD16^{+} monocytes. Phagocytosis measured using fluorescent beads and multiparametric flow cytometry. PBMCs were incubated overnight with stimulus and with beads for a further 3 hours (n=9). Each individual circle represents one individual subject. *P < 0.05, **P < 0.01, ***P < 0.001, and ****P < 0.0001 by two-way ANOVA or non-parametric Mann Whitney T-test.

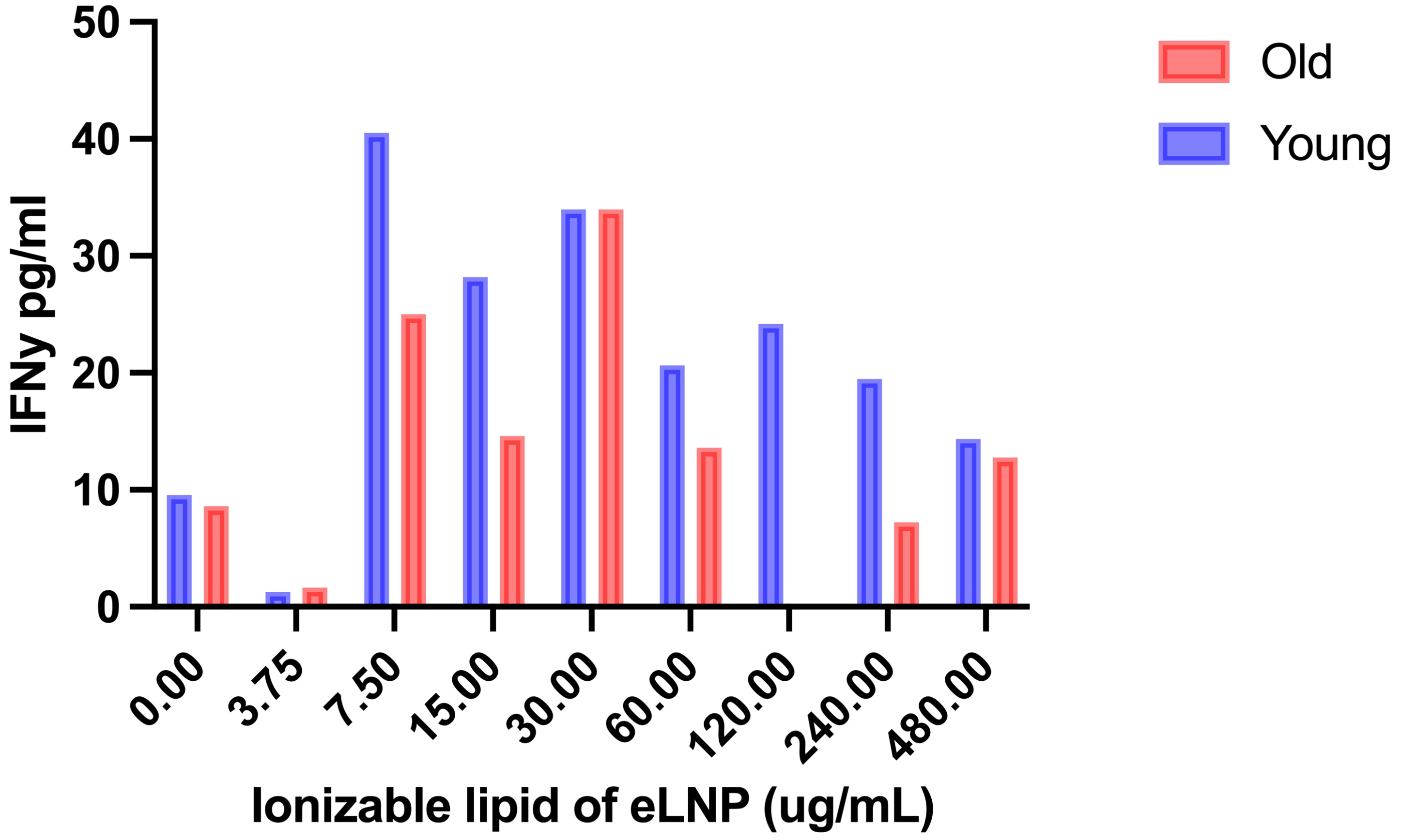

Supplemental figure 7: Optimal dose of eLNP

PBMCs were stimulated with eLNP in a twofold dilution to determine optimal dose for 24 hrs. Optimal concentrations of eLNP were selected based on median production of IFN-γ as measured by ELISA and an 85% or more survival rate, n=3. Bar represents the mean of all values.

Supplemental Table 1: Patient Recruitment and Enrollment

	Adults (n=11)	Older subjects (n=11)
Average age (range), y	30 (24-36)	73 (67-83)
Gender, male/female (female %)	4/7 (64)	5/6 (55)
Race, n		
White (non-Hispanic)	9 (82%)	11 (100%)
White (Hispanic)	2 (18%)	
Comorbidities, n	1 (9%)	9 (82%)
None	10 (91%)	2 (18%)
Arthritis	1 (9%)	5 (45%)
Hypertension		5 (45%)
Stroke		1 (9%)
Heart disease		1 (9%)
Medications		
Prescription	1 (9%)	6 (55%)
Over-the-counter	5 (45%)	8 (73%)

Supplemental Table 2: MDDC flow cytometry antibody list

Antigen	Clone	Fluorophore
CD3	HIT3α	PE-Cy7
CD56	5.1H11	PE-Cy7
CD19	HIB19	PE-Cy7
CD20	2H7	PE-Cy7
CD11c	BU15	BV605
HLA-DR	L243	APC-Cy7
CD40	5C3	APC
CD83	HB15e	PE-Cy5
CD86	IT2.2	FITC

Supplemental Table 3: PBMC flow cytometry antibody list

Antigen	Clone	Fluorophore
CD3	HIT3α	PE-Cy7
CD56	5.1H11	PE-Cy7
CD19	HIB19	PE-Cy7
CD20	2H7	PE-Cy7
CD11c	BU15	BV605
OX40L	Ik-1	BV711
41BBL	C65-485	BV786
CD14	M5E2	PE-Tx Red
HLA-DR	L243	AF700
CD16	3G8	BV650
CD1c	L161	BV605
CD303	201A	APC-Cy7
pIRF7	K47-671	APC
pTBK-1	J133-587	PE
STING	T3-680	BV421
CD141	AD5-14H12	FITC
CD80	2D10	PE-Cy5
PD-L1	MIH1	BUV395
CD16	3G8	BUV737
