# Supplementary material for: Lipid nanoparticles (LNP) induce activation and maturation of antigen presenting cells in young and aged individuals
Source: Commun Biol. 2023 Feb 17;6:188. doi: 10.1038/s42003-023-04555-1 (PMC9936473; doi:10.1038/s42003-023-04555-1)
Supplement: Supplementary file 4 — Reporting Summary [file 42003_2023_4555_MOESM4_ESM.pdf]

nature portfolio

Corresponding author(s): Elias Haddad

Last updated by author(s): 2022-23-JAN

Reporting Summary

Nature Portfolio wishes to improve the reproducibility of the work that we publish. This form provides structure for consistency and transparency in reporting. For further information on Nature Portfolio policies, see our Editorial Policies and the Editorial Policy Checklist.

Statistics

For all statistical analyses, confirm that the following items are present in the figure legend, table legend, main text, or Methods section.

n/a	Confirmed	
☐	☒	The exact sample size (*n*) for each experimental group/condition, given as a discrete number and unit of measurement
☐	☒	A statement on whether measurements were taken from distinct samples or whether the same sample was measured repeatedly
☐	☒	The statistical test(s) used AND whether they are one- or two-sided<br>*Only common tests should be described solely by name; describe more complex techniques in the Methods section.*
☐	☒	A description of all covariates tested
☐	☒	A description of any assumptions or corrections, such as tests of normality and adjustment for multiple comparisons
☐	☒	A full description of the statistical parameters including central tendency (e.g. means) or other basic estimates (e.g. regression coefficient) AND variation (e.g. standard deviation) or associated estimates of uncertainty (e.g. confidence intervals)
☐	☒	For null hypothesis testing, the test statistic (e.g. *F*, *t*, *r*) with confidence intervals, effect sizes, degrees of freedom and *P* value noted<br>*Give P values as exact values whenever suitable.*
☒	☐	For Bayesian analysis, information on the choice of priors and Markov chain Monte Carlo settings
☒	☐	For hierarchical and complex designs, identification of the appropriate level for tests and full reporting of outcomes
☒	☐	Estimates of effect sizes (e.g. Cohen's *d*, Pearson's *r*), indicating how they were calculated

Our web collection on statistics for biologists contains articles on many of the points above.

Software and code

Policy information about availability of computer code

Data collection	NA
Data analysis	FlowJo V10.8.1 and Graphpad Prism V9 were used to analyze all data sets

For manuscripts utilizing custom algorithms or software that are central to the research but not yet described in published literature, software must be made available to editors and reviewers. We strongly encourage code deposition in a community repository (e.g. GitHub). See the Nature Portfolio guidelines for submitting code & software for further information.

Data

Policy information about availability of data

All manuscripts must include a data availability statement. This statement should provide the following information, where applicable:

- Accession codes, unique identifiers, or web links for publicly available datasets
- A description of any restrictions on data availability
- For clinical datasets or third party data, please ensure that the statement adheres to our policy

The datasets generated during and/or analysed during the current study are available from the corresponding author on reasonable request.

Human research participants

Policy information about studies involving human research participants and Sex and Gender in Research.

Reporting on sex and gender	NA
Population characteristics	PBMCs were obtained from healthy donors age 18-80 with a mix of both males and females and different races.
Recruitment	Consenting adults were screened using a questionnaire determining their demographic information, medication usage, and co morbidities.
Ethics oversight	Martin Memorial Health Systems (Florida); the Institutional Review Boards at the institution approved all procedures, and all participants provided signed, informed consent

Note that full information on the approval of the study protocol must also be provided in the manuscript.

Field-specific reporting

Please select the one below that is the best fit for your research. If you are not sure, read the appropriate sections before making your selection.

☒ Life sciences ☐ Behavioural & social sciences ☐ Ecological, evolutionary & environmental sciences

For a reference copy of the document with all sections, see nature.com/documents/nr-reporting-summary-flat.pdf

Life sciences study design

All studies must disclose on these points even when the disclosure is negative.

Sample size	Sample sizes were not predetermined based on statistical methods, but were chosen according to the standards of the field. This is due to previous work utilizing these samples from this cohort and takes into account the heterogeneity of human samples.
Data exclusions	No data were excluded from this study
Replication	Reported results were replicated across multiple experiments with replicates generating similar results.
Randomization	Each donor was used for all experimental subgroups so autologous and heterologous controls for each human subject were included
Blinding	Luminex results from culture experiments were blindly analyzed; samples were blinded and given a number to keep from bias. The researcher running the assays and the person analyzing results were different.

Reporting for specific materials, systems and methods

We require information from authors about some types of materials, experimental systems and methods used in many studies. Here, indicate whether each material, system or method listed is relevant to your study. If you are not sure if a list item applies to your research, read the appropriate section before selecting a response.

Materials & experimental systems

n/a	Involved in the study	
☐	☒	Antibodies
☒	☐	Eukaryotic cell lines
☒	☐	Palaeontology and archaeology
☒	☐	Animals and other organisms
☒	☐	Clinical data
☒	☐	Dual use research of concern

Methods

n/a	Involved in the study	
☒	☐	ChIP-seq
☐	☒	Flow cytometry
☒	☐	MRI-based neuroimaging

Antibodies

Antibodies used	All antibodies are listed in supplemental information with supplier name, clone, and catalog number
Validation	Antibodies are used as per manufacturer's recommendation for concentration and titrated as neccesary

Flow Cytometry

Plots

Confirm that:

☒ The axis labels state the marker and fluorochrome used (e.g. CD4-FITC).

☒ The axis scales are clearly visible. Include numbers along axes only for bottom left plot of group (a 'group' is an analysis of identical markers).

☒ All plots are contour plots with outliers or pseudocolor plots.

☒ A numerical value for number of cells or percentage (with statistics) is provided.

Methodology

Sample preparation	Sample preparation listed in methods
Instrument	BD Fortessa
Software	FACS Diva for collection and data analyzed using FlowJo (v10)
Cell population abundance	Cell populations were determined from at least 1 million PBMCs stained and run by flow cytometry. Purity was assessed by staining for cell specific markers.
Gating strategy	Relevant gating strategies are shown in supplemental information

☒ Tick this box to confirm that a figure exemplifying the gating strategy is provided in the Supplementary Information.
